# Consistent individual differences and population plasticity in network-derived sociality: An experimental manipulation of density in a gregarious ungulate

**DOI:** 10.1371/journal.pone.0193425

**Published:** 2018-03-01

**Authors:** Paul P. O’Brien, Quinn M. R. Webber, Eric Vander Wal

**Affiliations:** 1 Department of Biology, Memorial University of Newfoundland, St. John’s, Newfoundland and Labrador, Canada; 2 Cognitive and Behavioural Ecology Interdisciplinary Program, Memorial University of Newfoundland, St. John’s, Newfoundland and Labrador, Canada; University of Lethbridge, CANADA

## Abstract

In many taxa, individual social traits appear to be consistent across time and context, thus meeting the criteria for animal personality. How these differences are maintained in response to changes in population density is unknown, particularly in large mammals, such as ungulates. Using a behavioral reaction norm (BRN) framework, we examined how among- and within-individual variation in social connectedness, measured using social network analyses, change as a function of population density. We studied a captive herd of elk (*Cervus canadensis*) separated into a group of male elk and a group of female elk. Males and females were exposed to three different density treatments and we recorded social associations between individuals with proximity-detecting radio-collars fitted to elk. We constructed social networks using dyadic association data and calculated three social network metrics reflective of social connectedness: eigenvector centrality, graph strength, and degree. Elk exhibited consistent individual differences in social connectedness across densities; however, they showed little individual variation in their response to changes in density, i.e., individuals oftentimes responded plastically, but in the same manner to changes in density. Female elk had highest connectedness at an intermediate density. In contrast, male elk increased connectedness with increasing density. Whereas this may suggest that the benefits of social connectedness outweigh the costs of increased competition at higher density for males, females appear to exhibit a threshold in social benefits (e.g. predator detection and forage information). Our study illustrates the importance of viewing social connectedness as a density-dependent trait, particularly in the context of plasticity. Moreover, we highlight the need to revisit our understanding of density dependence as a population-level phenomenon by accounting for consistent individual differences not only in social connectedness, but likely in other ecological processes (e.g., predator-prey dynamics, mate choice, disease transfer).

## Introduction

Phenotypic plasticity is when a trait changes across an environmental gradient through plasticity at the population, individual, or genetic levels [1]. The primary objectives associated with studying phenotypic plasticity are understanding if variance in a given trait exists, and, subsequently understanding potential sources of variance in the trait [2]. Variance at the population-level reflects changes in mean trait values across an environmental gradient, while individual-level plasticity occurs when individuals differ in their trait–environment relationship, i.e., an Individual–Environment interaction [1]. Similarly, if individual variation in plasticity is heritable, there is potential for trait evolution as environmental conditions change, i.e., a Genotype–Environment interaction [1]. Life-history, morphological, and behavioral phenotypes are therefore predicted to vary, i.e., display plasticity, across a range of environmental gradients, for example, thermal, predation, or population density gradients [3–5]. In this study, we examine among and within-individual behavioral consistency and plasticity in social network derived behavior as a function of changes in population density.

In the context of behavioral plasticity, behavioral reaction norms (BRNs) are an important tool for generating two important parameters: 1) the reaction norm slope, which corresponds to phenotypic plasticity; and 2) the reaction norm intercept, which corresponds to consistent individual differences in behavior, or animal personality [1,6]. The animal personality concept can be integrated within the conceptual framework associated with phenotypic plasticity using BRNs so behavioral consistency and plasticity can be quantified simultaneously [6]. Evolutionary correlations between within-individual differences in behavioral traits (reaction norm intercept) and plasticity (reaction norm slope) could result in environment-specific repeatability or heritability. For example, male great tits (*Parus major*) were more aggressive (reaction norm intercept) in areas with higher breeding densities (reaction norm slope), while both traits appeared to be heritable, suggesting the potential adaptive value of behavioral plasticity [7]. Meanwhile, quantifying social behavior in a BRN context could be highly relevant if the environmental gradient of interest is population density [4] because individuals may differ consistently in their social behavior, while some social behaviors are also density-dependent, and therefore likely display plasticity across a population density gradient.

Density dependence is a key ecological process that can influence population dynamics [8,9]. Changes in population density can, for example, influence body mass, fecundity, and survival [10,11]. While density can cause changes visible at the population-level, it is important to highlight the scale at which density acts [12] as well as behavioral phenotypes affected by density. Local population density, for example, can influence individual behavioral traits, including social aggregation [13] and fine-scale social interactions [14]. Population density can therefore affect an individual’s social environment and may affect both repeatability and plasticity of individual social phenotypes [15]. Understanding variance in individual social behavior and plasticity as a function of changes in population density is particularly apt because population density can influence sociality via competition [16–18] or through changes in group size and composition [13].

At the group, or population, level, social behavior can exhibit plasticity in response to changes in local density [19,20], where all individuals respond similarly to changes in density [1,6]. Conversely, within-individual variance in social behavior can be the product of changes in density. Specifically, individual social behavior can be density-dependent [21,22] or independent of density [23], highlighting the importance of phenotypic plasticity in the context of an individual’s social environment [24]. Individual differences in social behavior can be consistent across time and contexts [25], and may meet the criteria for animal personality, i.e., consistent individual differences in behavior across time and contexts [26,27]. Behavioral variation associated with animal personality and plasticity may be important for individuals to adapt to changes in population density [28]. For instance, at low local density, selection favoured fast exploring great tits, *Parus major*, while at high density, selection favoured slow exploring birds, presumably because temporal variation in local density selects for a range of personality types [4].

Elk, *Cervus canadensis*, are gregarious ungulates that exhibit sexual segregation outside of the breeding season [29]. Elk inhabit open areas and presumably adopt group living as an antipredator defense; however, elk also exhibit variation in local densities and habitat use [30]. Female elk typically raise offspring in social groups [29] to reduce calf predation risk via dilution, i.e., per capita predation risk decreases as group size increases and to reduce predation via detection effects, i.e., larger groups increase the ability to detect predators [31]. The relationship between density and some social behaviors, e.g., rate of encounter, for female elk is non-linear [19,20], likely because there is an optimal density beyond which competition for resources is too high and social aggregation becomes too costly. Males show lower social affiliation than females and social interactions tend to be more aggressive than affiliative as males establish dominance hierarchies prior to the breeding season [29]. However, pre-rut and rut hierarchies are not always the same and the consistent maintenance of hierarchies may be required [30]. Specifically, male vigilance is primarily directed toward conspecifics rather than potential predators [31].

The present study builds on previously published work from this system [20], where group-level encounter rates were compared across densities. Our prior work in this system suggests that, at the population level, mean phenotypic trait values change as a function of population density, however, the possibility for an Individual–Environment interaction remains untested. Our objective was to quantify individual social connectedness, as measured by metrics of social network centrality, within a behavioral reaction norm framework to assess the repeatability and plasticity of individual social connectedness as a function of experimentally manipulated population density (for similar examples see Wilson et al. [32] and Krause et al.[33]). Using social network analyses, we quantified three measures of individual social centrality, each of which reflected either direct or indirect connections among group members: 1) eigenvector centrality (direct and indirect connections); 2) graph strength (direct connections); 3) and degree (direct connections) [34]. We tested two hypotheses:

1. We hypothesized that among-individual differences in social connectedness in response to density would vary for males versus females given behavioral differences that exist outside of the breeding season (S1 Table) [18]. Our individual level predictions are based on population level observations [19,20]. First, we predicted (P1a) that, for females, social connectedness would be highest at an intermediate density because population level encounter frequency is highest at intermediate density [19,20] and we presume there should be an optimal density beyond which the individual costs of group living (i.e., competition for resources, disease transfer, etc.) outweigh the benefits for females. Second, we predicted (P1b) that, for males, social connectedness would increase with increasing density because population level encounter frequency increases linearly with increasing density [20] and given that males typically form smaller groups outside of the breeding season [13], increasing density should result in more social connections.
2. We hypothesized that among-individual social connectedness in elk would be repeatable and plastic across densities (S1 Table). First, we predicted (P2a) that, if repeatable, individuals would exhibit consistent differences in reaction norm intercepts, such that individuals with higher values of social connectedness will maintain higher social connectedness values across densities because elk display social flexibility among-individuals in response to changes in population density [19,20]. Second, we predicted (P2b) that, if individuals vary in their ability to adapt to changes in population density [1,35], then within-individual plasticity will exist, such that individuals will exhibit differences in reaction norm slopes.

## Materials and methods

### Data collection

We collected data from a small herd of captive-born adult elk, *Cervus canadensis* (14 females, age 4.3–10.9 years and 11 males, all aged 6 years) at an experimental farm, in summer 2007, i.e., outside of the breeding season. Elk were located at the Specialized Research Centre field plots of the Western College of Veterinary Medicine in Saskatchewan, Canada (S1 Fig; See Vander Wal et al. [20] for aerial photograph of field plots). All research was approved by the University of Saskatchewan Animal Care committee under protocol number 2006006. Typical husbandry practices keep elk sexually segregated. Therefore, the captive elk herd was separated by sex into groups of male and female elk that did not interact during our study. Male and female groups were similar in size to those of wild elk [13,36,37]. Prior to our study, males had antlers removed to prevent injury and females were without calves. To prevent elk from becoming habituated to humans we handled animals as little as possible. As such, elk were only handled twice during the study to apply and remove Sirtrack Proximity Logger radio-collars (Sirtrack, Havelock North, New Zealand) at the beginning and end of the experiment, respectively. We fit elk with collars by corralling animals into a livestock chute and immobilizing them in a modified cattle squeeze (for details see Vander Wal et al. [20]). Within the female group, two collars malfunctioned after deployment, so we did not include these individuals for subsequent analysis. Thus, for the purposes of our analysis, we refer to the female herd as being comprised of 12 individuals.

Each Sirtrack collar was programmed to activate and record encounter data (i.e., frequency and duration of encounters in seconds) whenever one collar came within 1.4 m (SD ± 1.00 m) of another, approximately one body length [20,38]. Collars deactivated when a pair was separated for >30 s and at a distance of 1.8 m (distance of collar deactivation 1.98 m, SD ± 1.60 m). Encounters were considered fine-scale proximal associations, not interactions nor contacts as defined by Whitehead and Dufault [39]. Due to inherent variation associated with ultra-high frequency (UHF) signal strength, proximity collars do not perfectly record mirrored encounters, i.e., within the dyads, the information from one collar may vary slightly from the other animal in the pair. This technical collar bias was corrected for as described by Boyland et al. [40]. Here, our asymmetrical matrix of encounter data was standardized using the percent pairwise differences between the most deviant collar and every other collar to maximize the correlation between the upper and lower triangles of the matrix. This process was repeated for each replicate within treatments [40].

### Experimental design

Elk husbandry practices mirror elk behavior in the wild and elk typically exhibit sexual segregation outside the breeding season [29,41,42], so we provided separate enclosures for males and females. We then randomized and replicated treatments for each sex at three densities: low, medium, and high. Enclosures for the female herd (n = 12) were 19.6, 13.4, and 9.8 ha (densities: 0.71 elk/ha, 1.05 elk/ha, and 1.43 elk/ha, respectively). Enclosures for the male herd (n = 11) were 13.4, 9.8, and 6.7 ha (densities: 0.75 elk/ha, 1.02 elk/ha, and 1.49 elk/ha, respectively). The same enclosures were used for both replicates at each density (S1 Fig). Male and female herds were exposed to each density treatment twice, for a total of six experimental units per sex (three densities by two replicates for both males and females). These represent a range of densities of elk in the wild [13,43,44], particularly at this fine scale (i.e., examining within-group interactions). Our experiment represents one way in which density can be manipulated without affecting the social structure, i.e., changing the area occupied. Our method is similar to the approach used by Wilson et al. [32], where the water level, and thus area occupied by fish, was altered to address changes in social networks across densities. Other means of manipulating density exist, e.g., adding or removing individuals; however, such methods fail to preserve existing social structure across treatments and replicates, thus confounding the experiment. See Vander Wal et al. [20] for more details.

Elk were herded between treatments without direct handling. Treatments and replicates ran for seven days and the order of treatments was randomized to reduce bias that may result from change in day length or forage condition over the experimental period (see S2 Table for information on treatment order and enclosure design). Food was available *ad libitum* to all individuals during each treatment; thus, food competition during our experiments was likely negligible. To account for possible confounding effects of handling, we removed days where herds were collared or moved among corrals from subsequent analyses. All networks were therefore composed of only 5 days of data. To minimize observer bias, we were blind to the identity of individual animals during analysis.

### Social network analysis

We constructed undirected, weighted social networks for each of three density treatments and two replicates per density treatment, based on dyadic encounters between uniquely identifiable individuals for both male and female herds. Networks were undirected because without direct visual observation it is impossible to determine which individual initiated a given encounter. We weighted networks by frequency of encounter as opposed to binary as an indicator of the strength of encounters among dyads. Networks consisted of nodes, i.e., individuals, and edges, i.e., connections between individuals, and were constructed using the *igraph* [45] package in R (version 3.4.3; [46]).

Based on frequency of encounter data among elk, we calculated three individual-level social network metrics as proxies for individual social connectedness: eigenvector centrality, graph strength, and degree. Eigenvector centrality corresponds to the first eigenvector of an adjacency matrix, and eigenvector centrality values reflect measures of an individuals’ direct and indirect connections; thus, an individual with high eigenvector centrality is connected to many individuals that also have many connections [45,47], but see Bonacich [48] for details on calculation of eigenvector centrality. Measures of social centrality that incorporate direct and indirect connections, such as eigenvector centrality, can be important regarding transfer of information or pathogens [47]. Graph strength accounts for direct associations among individuals and is the sum of all weighted associations of an individual [45] and in this case accounts for the frequency of encounters rather than the duration. Strength is a common network metric that has been found to be repeatable in a number of taxa [33,49–52]. Degree represents the total number of individuals the focal individual associates with and is a binary metric, meaning it only accounts for presence or absence of encounters among individuals [45] and can be used as a measure of gregariousness [50]. Graph strength and degree are direct measures of sociality because they only account for the focal individual’s immediate connections. Although subtle, individuals may differ in their indirect and direct social connections [47].

To ensure individual social network metrics, i.e. eigenvector centrality, graph strength, and degree, were non-randomly distributed we conducted two types of network randomization procedures. First, we employed a randomization technique where social association matrices were permutated with 1,000 iterations and network metrics were re-calculated at each permutation [53]. Permutations were computed based on social association matrices as opposed to the data stream, i.e. frequency of associations generated from proximity collars, because all animals in our study were collared and proximity collars collect data continuously throughout the experiment. We considered observed network metrics non-random if the mean observed value fell outside the 95% confidence interval of the random distribution [54]. For each network metric, we generated random distributions for each replicate (1 or 2) by density (low, medium, or high) by sex (male or female) for a total 36 randomizations (S2–S7 Figs).

Second, we randomized network metrics within our modeling framework (see below) and re-calculated repeatability for each network metric at each of 1,000 iterations (see below for details on calculating repeatability). We generated random distributions of repeatability values (and the posterior distributions random effects used to calculate repeatability) from the best model (see statistical analyses section) and compared the observed repeatability value to the distribution of randomly generated repeatability values (S8 Fig). We considered observed repeatability non-random if the mean observed value fell outside the 95% confidence interval of the random distribution. Our randomization procedures allowed us to assess whether our observed network metrics reflected biologically meaningful social structure as well as values of repeatability or, alternatively, whether they were the outcome of random combinations of individual connections.

### Statistical analyses

We estimated repeatability of each social network using a Bayesian framework (Package 'MCMCglmm'; [55]). Bayesian analysis requires the specification of prior distributions for unknown parameters [56], but when prior knowledge is minimal, uninformative priors are recommended [55]. Thus, for both random effects (*G*) and residuals (*R*) we coded variance (*s*^*2*^) in our priors as *s*^*2*^/2 and degree of belief (nu) as one. To ensure our results were not biased by priors, we re-ran all models with variance coded as *s*^*2*^/4 and *s*^*2*^/6 to ensure our results were not dependent on the selected priors. All models were fit with Gaussian error structure and we assessed normality prior to analyses. We assessed autocorrelation for Markov Chain Monte Carlo (MCMC) chains for models using the ‘autocorr’ function to ensure autocorrelation was < 0.1 [55]. To ensure MCMC chains were uncorrelated we ran conservative MCMC chains of 1,300,000 iterations, a thinning length of 1,000, and a burn-in of 300,000. Model convergence was confirmed visually.

To test the effect of density on individual connectedness, we employed six competing Bayesian univariate mixed-models separated by sex (S3 Table). We generated models for each of three social network metrics as the response variable, for a total of 36 models, each including 6 total values per individual for each metric (i.e., 2 replicates by 3 treatments; S2 Table; see S1 Appendix for information on replicates). Our models included: 1) a null model with only an intercept; 2) an intercept only model, with ID as a random intercept; 3) density as a fixed effect, with ID as a random intercept; 4) quadratic density, i.e., density + density^2^, to account for possible non-linear trends, as fixed effects with ID as a random intercept; 5) quadratic density as fixed effects with ID and density as random intercept and slope for each individual, respectively; and 6) quadratic density as fixed effects with ID and quadratic density as random intercept, slope, and curvature for each individual, respectively. For each model, we considered covariance in all specified random components. Incorporating density as a fixed effect in our models describes the *mean* change of a social network metric across changes in density, while incorporating density and density^2^ as random effects allowed us to model *individual* deviation from the fixed effect mean, i.e., within-individual differences in linear and non-linear reaction norm slopes, respectively [57].

We calculated Deviance Information Criteria (DIC) for each model to determine parsimony of candidate models following Murtaugh [58] (for examples using stepwise regression see Wilson et al. [56] and Nussey et al. [1]). We considered models with ΔDIC < 2 as indistinguishable [59,60]. For cases with ΔDIC < 2, we selected the candidate model containing the highest number of fixed effects as the best-fitting model, however, in some instances simpler but indistinguishable models, i.e., ΔDIC < 2, may exist. The most complex model was chosen over simpler, indistinguishable models to avoid loss of information.

### Repeatability and plasticity in behavioral responses

To determine if individual measures of social connectedness were consistent across densities, we calculated repeatability (*r*) using the variance (*s*^*2*^) of random effects:

$$r=\frac{s_{among}^{2}}{s_{among}^{2}+s_{within}^{2}}$$

where *s*^*2*^_*among*_ is the among-individual variance (elk ID) and *s*^*2*^_*within*_ is the within-individual variance (residual). Highly repeatable behaviors have values close to 1, while behaviors with low repeatability do not differ from 0 [61]. We therefore estimated changes in individual social behaviors across a density gradient [6] to ascertain if changes in sociality are density-dependent. Repeatability estimates and associated 95% credibility intervals were calculated based on the posterior distribution for the most parsimonious model based on the process described above. Among-individual variance was partitioned among random effects for selected models to determine the contribution of each source to the calculated repeatability estimates. Following Wilson et al. [62] and Krause et al. [33] we also conducted a rank-order repeatability analysis to confirm the above repeatability analyses (see S2 Appendix). To our knowledge, this is the first study to assess repeatability of social network metrics derived from proximity detecting collars, and, as a result, inherent collar bias may exist in our repeatability estimates. To address this issue, we compared social network metrics generated based on collars deployed on individual elk in this study to the same collars deployed on elk in a different study system (Vander Wal et al. [38]; see S3 Appendix for details). All statistical analyses were conducted in R [46].

## Results

**Fig 1:**
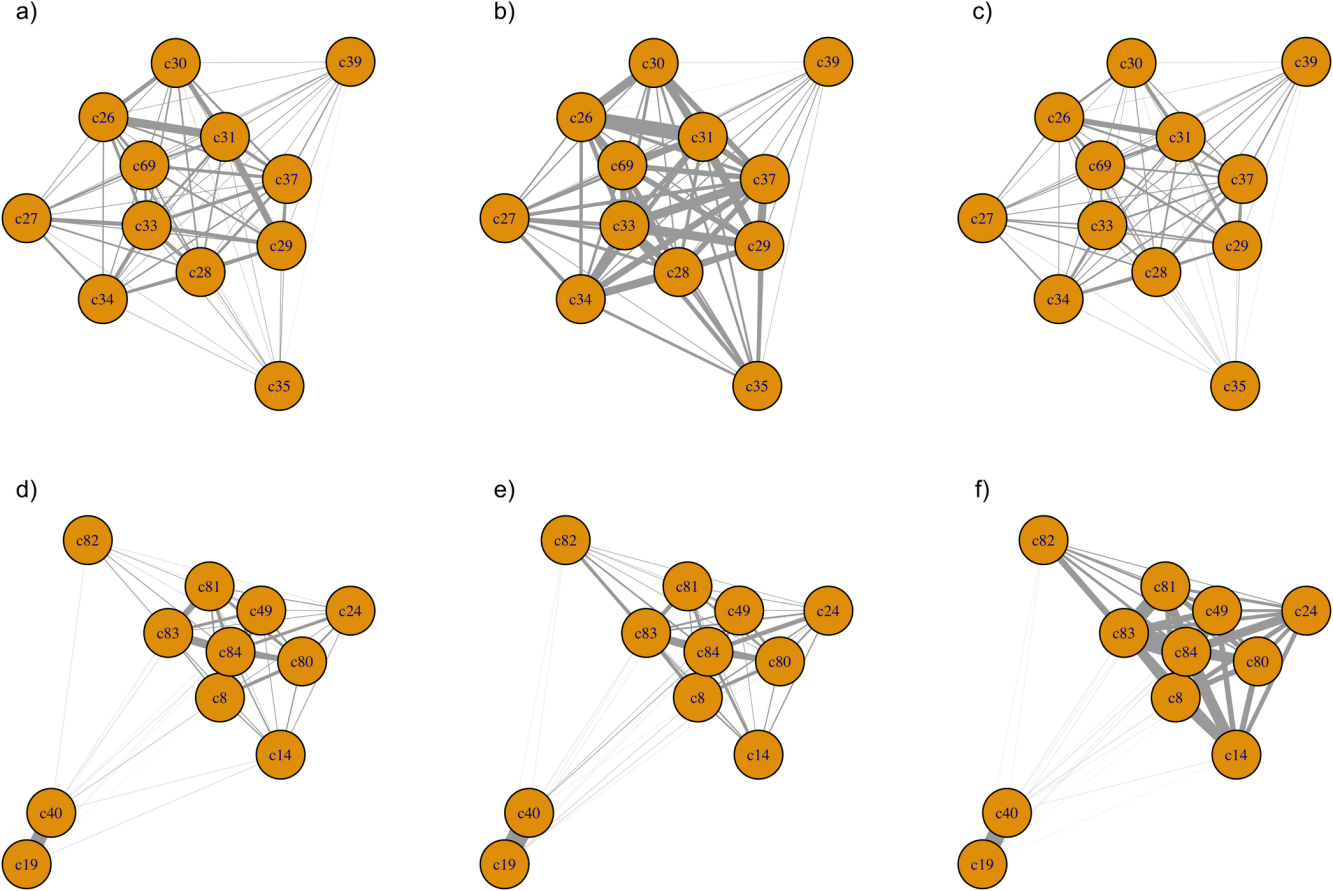
**Illustrative social networks of female (a, b, c) and male (d, e, f) captive elk (*Cervus canadensis*).** Nodes represent individual elk and edge thickness represents association strength. Networks show associations at (a) and (d) low density; (b) and (e) medium density; and (c) and (f) high density; for females and males, respectively.

**Fig 2:**
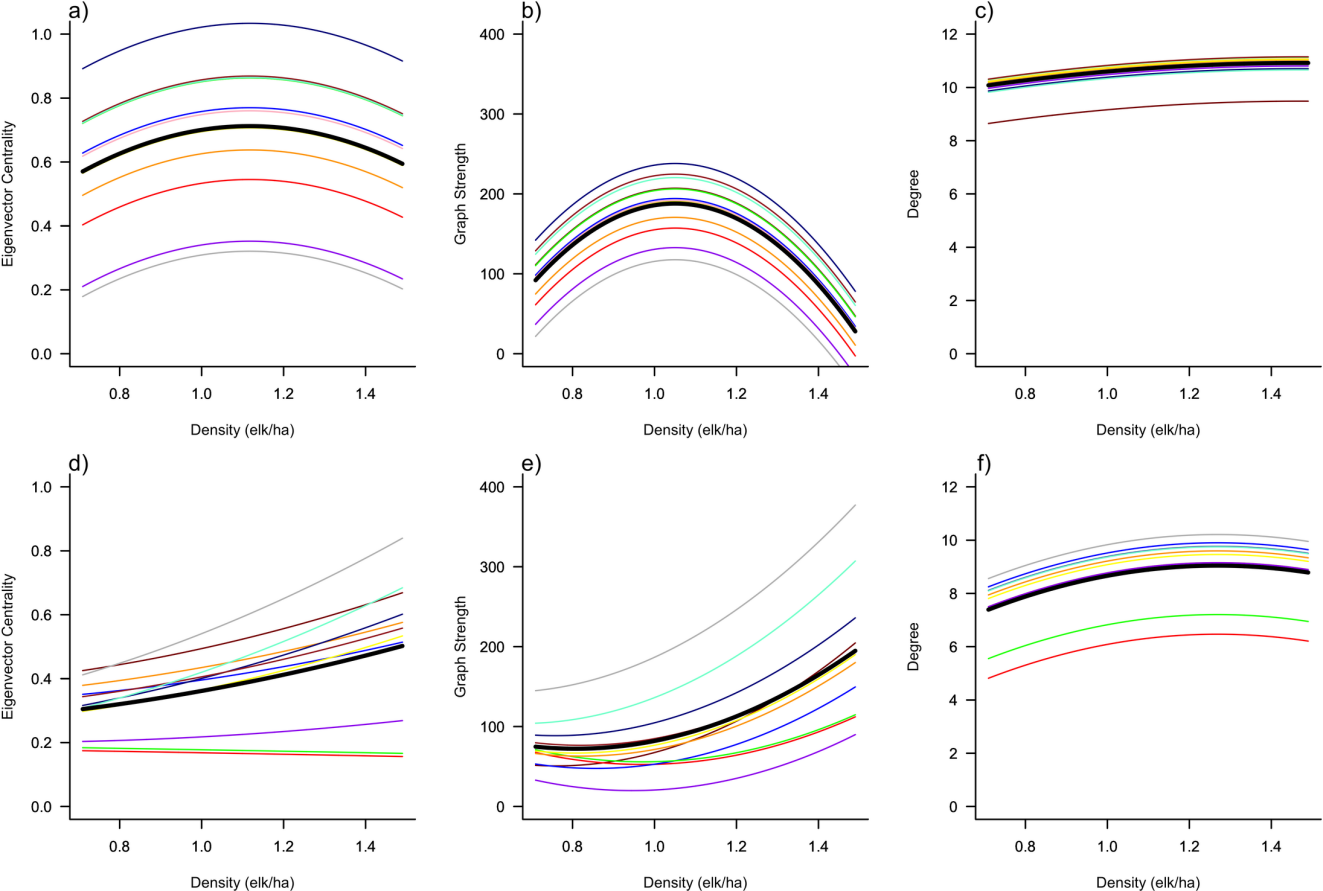
Variation in (a) and (d) eigenvector centrality; (b) and (e) graph strength; and (c) and (f) degree; for females and males, respectively, illustrating consistent individual differences (in colour) and population mean (in black) response to changing conspecific density for female (n = 12) and male (n = 11) captive elk in Saskatchewan (2007). Curves were generated using Bayesian model predictions based on the model generated relationship between density and network metrics across two replicates for each density.

**Fig 3:**
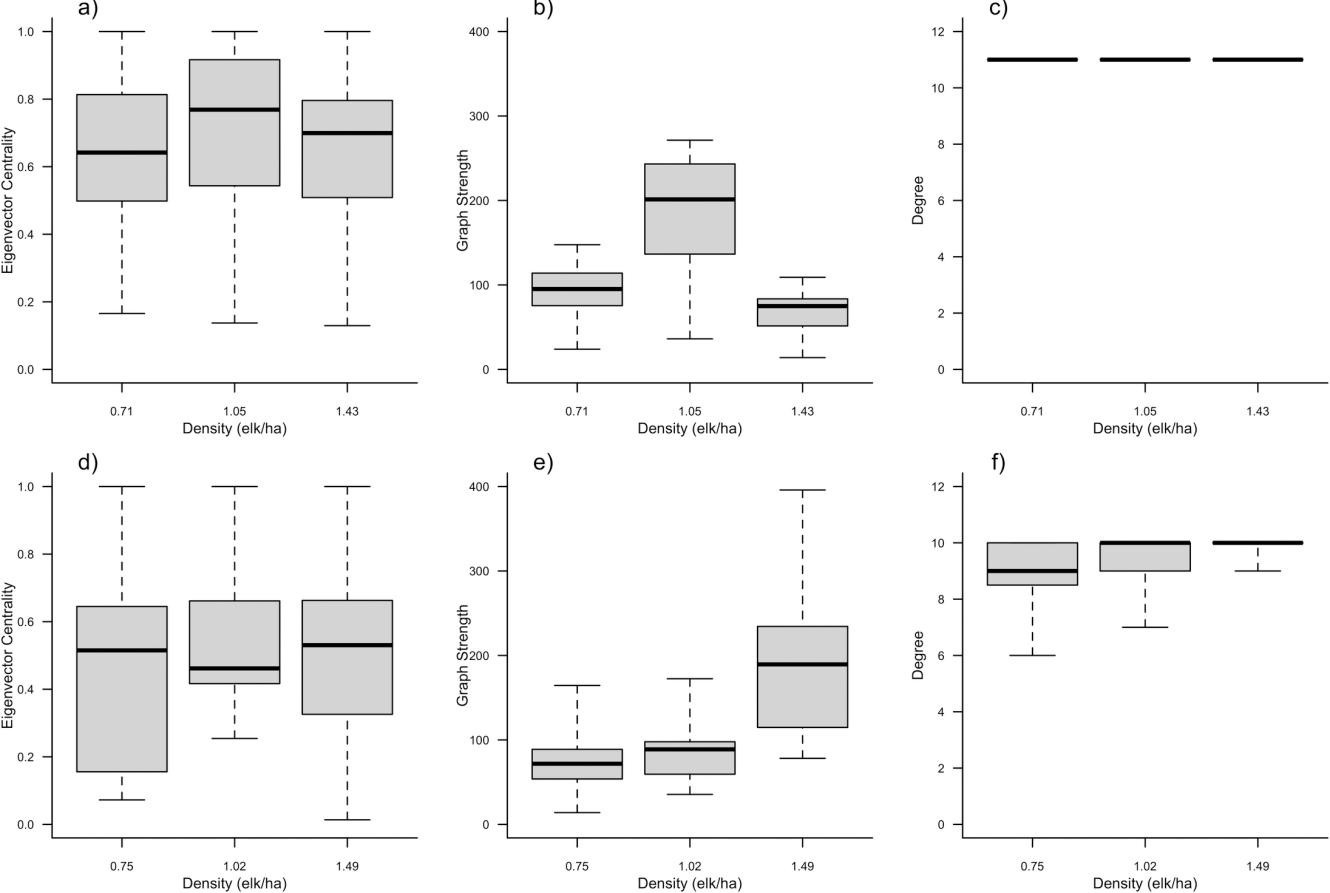
**Population-scale boxplots of median, 25% and 75% quartiles, and 95% confidence intervals (“whiskers”) showing population response to changing conspecific density for female (a, b, c; n = 12) and male (d, e, f; n = 11) captive elk (*Cervus canadensis*) in Saskatchewan (2007).** (a) and (d) are eigenvector centrality; (b) and (e) are graph strength; and (c) and (f) are degree; for females and males, respectively. Letters, i.e., ‘a’ and ‘b’, indicate significant differences between values of social network metrics at each density. For instance, any two boxes within a plot that share the same letter were not significantly different. Note, (c) there is no variance in degree because all females have identical values for degree, i.e., all individuals had social encounters with all other individuals during our density treatments.

The frequency of encounters for females was highest at an intermediate density and increased with increasing density for males as illustrated by the thickness of the edges (Figs 1 and 2). The two groups differed in how density affected centrality. For the female group, individual and population-level eigenvector centrality and graph strength were highest at intermediate density (Table 1, Fig 3A and 3B), while female degree did not differ across densities (Table 1, Fig 3C). For the male group, individual and population-level eigenvector centrality and graph strength increased with increasing density (Table 1, Fig 3D and 3E), while degree only increased slightly (Table 1, Fig 3F).

**Table 1:** Mean and standard deviation social network metric values for male and female elk (*Cervus canadensis*) groups for each density treatment and replicate. Note, each individual had six measures of each network metric which were used in subsequent analyses.

		Males			Females		
Density Treatment	Replicate	Eigenvector Centrality	Graph Strength	Degree	Eigenvector Centrality	Graph Strength	Degree
Low	1	0.22 (± 0.38)	63.4 (± 30.8)	7.5 (± 2.0)	0.70 (± 0.25)	123.8 (± 43.8)	11 (± 0)
Low	2	0.40 (± 0.32)	84.7 (± 60.8)	7.8 (± 2.0)	0.44 (± 0.31)	58.8 (± 38.6)	9.2 (± 2.1)
Medium	1	0.46 (± 0.30)	93.4 (± 48.2)	8.6 (± 1.4)	0.74 (± 0.26)	183.5 (± 67.2)	11 (± 0)
Medium	2	0.28 (± 0.36)	75.9 (± 37.4)	8.9 (± 1.2)	0.68 (± 0.31)	192.4 (± 90.1)	10.3 (± 0.98)
High	1	0.50 (± 0.32)	235 (± 132.7)	8.7 (± 1.4)	0.60 (± 0.25)	79.1 (± 33.5)	10.8 (± 0.39)
High	2	0.50 (± 0.31)	156.4 (± 74.8)	8.9 (± 1.2)	0.67 (± 0.27)	57.3 (± 22.7)	11 (± 0)

For females, eigenvector centrality, graph strength, and degree were best represented by M_4_ (Table 2) with density and density^2^ as fixed effects and only ID as a random effect, however; ΔDIC < 2 included the null model for degree. M_6_ (Table 2) best represented male eigenvector centrality and graph strength, while male degree was best represented by M_4_ (Table 2); however, ΔDIC < 2 also included the null model for male eigenvector centrality (Table 2; for summary of fixed and random effects see S4 Table; for modelled predictions see Fig 2).

**Table 2:** Repeatability values of individual eigenvector centrality, graph strength, and degree from six competing models for female and male elk (*Cervus canadensis*). Repeatability values for the most complex models with ΔDIC < 2 are emboldened with 95% credible intervals in brackets (see S3 Table for mathematical formulas of each model).

**Females**									
**Model**	**Fixed effects**	**Random effects**	**Centrality**		**Strength**			**Degree**	
			**ΔDIC**	**Repeatability**	**ΔDIC**		**Repeatability**	**ΔDIC**	**Repeatability**
M_1_	~1	-	60.74	**-**	77.48		-	**0.83**	-
M_2_	~1	ID	4.53	0.63 (0.42–0.85)	70.90		0.23 (0.07–0.47)	4.91	0.12 (0.03–0.28)
M_3_	~Density	ID	5.27	0.61 (0.40–0.83)	70.96		0.24 (0.07–0.47)	**0**	**0.14 (0.04–0.3)**
M_4_	~ Density + Density^2^	ID	**0**	**0.65 (0.42–0.84)**		**0**	**0.51 (0.25–0.73)**	**1.54**	**0.13 (0.04–0.3)**
M_5_	~ Density + Density^2^	ID+ Density	3.88	0.64 (0.45–0.84)	4.86		0.56 (0.36–0.79)	6.31	0.24 (0.1–0.46)
M_6_	~ Density + Density^2^	ID+ Density + Density^2^	8.98	0.67 (0.52–0.87)	10.58		0.63 (0.47–0.86)	10.66	0.33 (0.2–0.58)
**Males**									
**Model**	**Fixed effects**	**Random effects**	**Centrality**		**Strength**			**Degree**	
			**ΔDIC**	**Repeatability**	**ΔDIC**		**Repeatability**	**ΔDIC**	**Repeatability**
M_1_	~1	-	**1.64**	**-**	75.19		-	54.81	-
M_2_	~1	ID	2.37	0.17 (0.05–0.38)	66.15		0.28 (0.1–0.54)	16.12	0.53 (0.3–0.79)
M_3_	~Density	ID	**0**	**0.17 (0.04–0.4)**	24.39		0.45 (0.23–0.71)	5.25	0.59 (0.36–0.83)
M_4_	~ Density + Density^2^	ID	2.06	0.17 (0.04–0.39)	20.63		0.47 (0.23–0.73)	**0**	**0.61 (0.37–0.83)**
M_5_	~ Density + Density^2^	ID+ Density	2.03	0.27 (0.12–0.5)	6.23		0.65 (0.44–0.88)	4.36	0.65 (0.44–0.87)
M_6_	~ Density + Density^2^	ID+ Density + Density^2^	**1.26**	**0.41 (0.26–0.69)**	**0**		**0.69 (0.19–0.88)**	9.50	0.70 (0.49–0.88)

**Fig 4:**
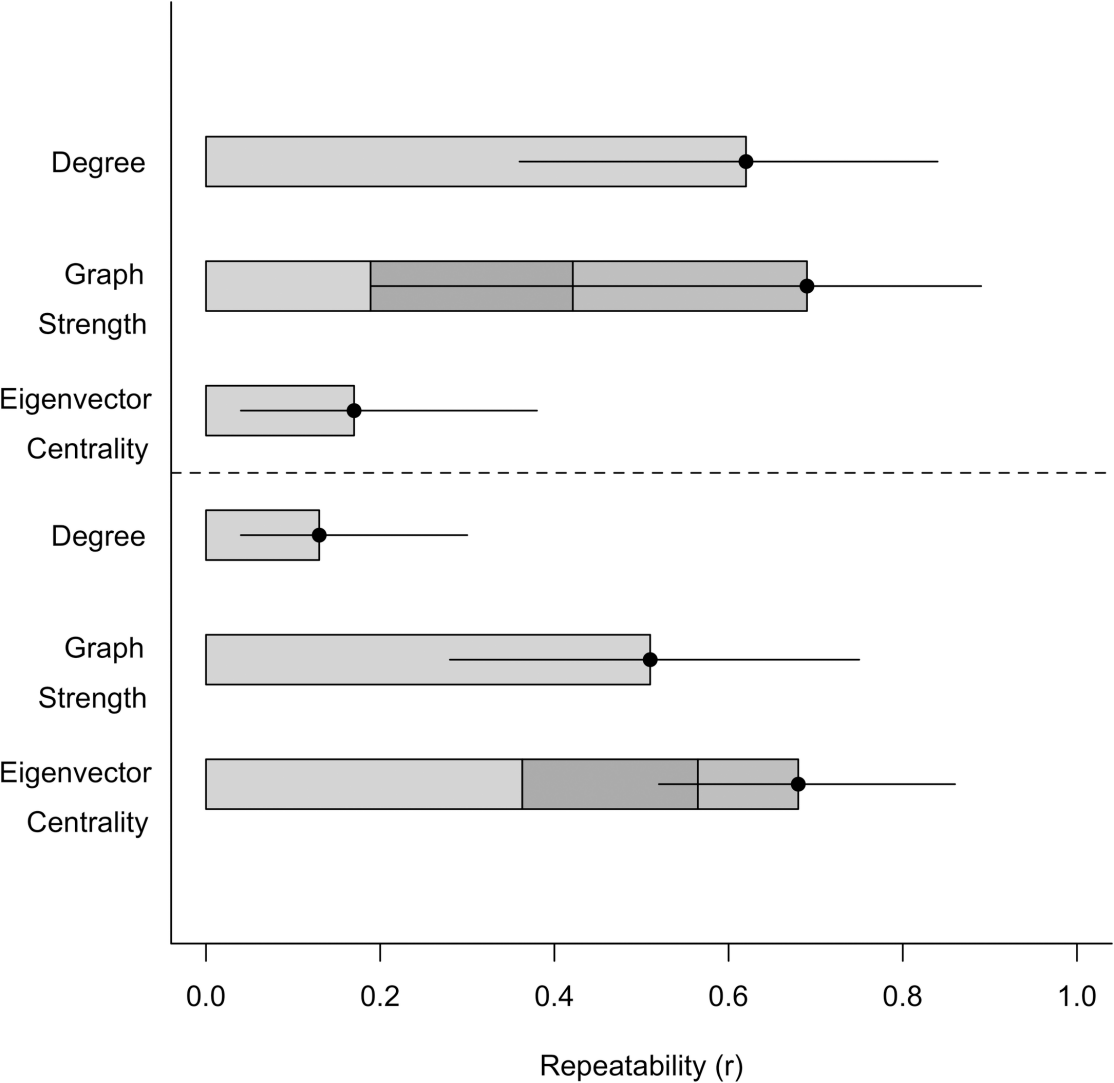
**Repeatabilities (*r*) as indicated by the full bar length with lower and upper 95% credible intervals for each social network metric for male (top) and female (bottom) elk (*Cervus canadensis*) in Saskatchewan (2007).** Among-individual variance for each repeatability value is partitioned among random effects: ID (light grey), density (dark grey), and density^2^ (medium grey) for the most complex, best-fitting models (See Table 2) to illustrate the contribution of each factor to the repeatability.

Individual differences in female social network metrics were repeatable: for eigenvector centrality, *r* = 0.65 (0.44–0.86), graph strength, *r* = 0.51 (0.25–0.73), and degree, *r* = 0.13 (0.05–0.32) (Table 2, Fig 4). Individual differences in male network metrics were repeatable: for graph strength, *r* = 0.69 (0.19–0.88), degree, *r* = 0.62 (0.4–0.83), and for eigenvector centrality, *r* = 0.41 (0.26–0.69) (Table 2, Fig 4). These repeatability results were qualitatively similar to our rank-order repeatability analyses (S5 Table). Individual differences in social network metrics were repeatable for males and females, however, the repeatability estimate for female degree appears to be lower than other metrics. For the male centrality model, the null model, i.e., the model with no random effects and therefore no conditional variance and no repeatability, is ΔDIC < 2 from the top model in the candidate set. This lends uncertainty to our repeatability estimates for this metric because our inference from the repeatability estimates and DIC are conflicting (Table 2). Individual variation was partitioned among random effects for repeatability estimates (Fig 4). Both male and female groups exhibited consistent individual differences in social centrality across density, but responses to density were the same for all individuals, i.e., there was no personality-dependent plasticity (Fig 2). Our comparison of social network metrics derived from the same proximity collars across study systems indicates no correlations (S7 Table), suggesting collar bias likely had little influence on our estimates of repeatability (S3 Appendix).

## Discussion

We found that three measures of individual social connectedness were density-dependent, where values of connectedness varied as a function of density for both male and female groups of elk. The observed effect of density varied for males and females, but similarly resulted in consistent individual differences in measures of social connectedness among individuals for male and female groups as well as population-level behavioral plasticity. Moreover, density dependence in male eigenvector centrality and graph strength exhibited within-individual plasticity across a density gradient, although we did not observe a similar trend for females. This diversity may stem from sex-based differences in social connectedness for elk and, possibly, the relative importance of direct vs. indirect social associations. Our findings highlight the importance of understanding how density dependence influences consistent individual differences in behaviors, such as social connectedness, as well as plasticity.

### Sex-based differences in social connectedness

Prior work in this system suggests that elk display sex-specific population-level differences in social encounter rate [20], but here, we observed similar trends at the within-individual level, thus supporting our predictions (P1a and b). In the female group, we found a non-linear relationship between social centrality and density both within- and among-individuals, such that centrality for individual female elk was highest at an intermediate density. At high densities, when high encounter rates are likely, we observed a decrease in encounter rates, suggesting a cost associated with connectedness at high density. Reduced association at high density could be an adaptive behavioral tactic to avoid increased competition [63] or risk of acquiring parasites and pathogens [64]. For wild elk, female aggregation at intermediate densities could influence fitness, especially if groups are large enough to increase predator vigilance, but small enough to reduce costs associated with competition or parasitism.

In the male group, we found a positive, linear relationship between social connectedness and density both within- and among-individuals, potentially because males differ in their vigilance behavior compared to females. Male vigilance is primarily directed toward conspecifics rather than predators [31], suggesting that increases in connectedness for males at high densities could reflect increased competition via aggregation. Connectedness may have increased with density because males were establishing a pre-rut dominance hierarchy. Male elk increase dominance behavior prior to, and throughout, the rut [63], which suggests density may affect dominance hierarchies. One hypothesis stemming from this finding is that social connectedness and dominance are linked [65], and that dominant elk are more central because they exert dominance over many conspecifics.

### Personality, plasticity, and reaction norms

Our results illustrate how individual social connectedness responds to changes in density, such that elk exhibited among-individual plasticity in the three social network metrics in response to changes in density. Network metrics were also highly repeatable across densities. As we predicted (P2a), the most central individuals at one density remained the most central individuals across densities, i.e., consistent between-individual differences in reaction norm intercepts, thus confirming the presence of animal personality. These results were corroborated by our rank-order analyses (S5 Table). However, we found limited support for our prediction (P2b) of within-individual variance in plasticity where we predicted that reaction norm slopes would differ. In general, we found that all individuals tended to respond in a similar manner to changes in population density via similar within-individual reaction norm slopes. However, individuals maintained rank-order differences in social network metrics as density changed (S5 Table), suggesting the most social elk at one density remained the most social elk at other densities. Although reaction norm slopes rarely crossed, i.e., displayed personality-dependent plasticity [2], we observed some subtle differences in reaction norm slopes for individual male, but not female, eigenvector and graph strength (Fig 2). Our reaction norms, in combination with evolutionary theory and empirical data, suggest that personality traits, including social connectedness, may be under density-dependent selection [66]. For instance, in great tits, individuals with fast exploring phenotypes were favoured at low density, while individuals with slow exploring phenotypes were favoured at high density [4]. Variation in reaction norm intercepts of social connectedness may also be a result of differential selection due to the costs and benefits associated with connectedness at different densities. Changes in density could therefore favour flexibility of social phenotypes in elk as well as other social vertebrates.

### Differences in measures of connectedness

We also found sex-specific differences in individual variation and the relative importance of graph strength and degree (direct measures) vs. eigenvector centrality (direct and indirect measure). Individual values of eigenvector centrality were more variable for females, possibly because females rely on group members for information. For free-ranging elk, this could be information about predators or resources. The large between-individual variation in eigenvector centrality among female elk illustrates how females may share information with group-members and, in general, maintaining higher levels of connectedness may be adaptive. For males, individual-level eigenvector centrality and graph strength were highly variable, particularly at high densities. One explanation could be that centrality and strength vary among individual males via direct and indirect aggressive interactions. For example, some males may generally be more aggressive, resulting in some dyads being more competitive with one another than other dyads. It is also possible that social association occurred as a function of unmeasured factors, such as relatedness. The sex differences we observed in measures of social connectedness may be linked to species-specific variation in ecological or behavioral factors among sexes, for example, fission-fusion dynamics and/or seasonal variation in social aggregation.

Understanding proximate costs and benefits associated with direct and indirect social connections is important because these differences could lead to variation in social dynamics or processes at the population level [15], especially if connectedness influences fitness. Strong direct social connections are associated with higher offspring survival [67] and reproductive success [68,69]. Indirect connections are also important, particularly regarding transfer of information or disease [47]. Although both information and disease transfer can be direct and indirect [70], indirect connections may play a significant role in information about predation risk. Indirect social connections can also influence pathogen dynamics, for example, highly connected individuals may be more likely to acquire and transmit pathogens [33,71]. The relationship between individual social behavior and pathogen dynamics could be important for elk because they host a number of socioeconomically relevant infectious diseases (e.g., bovine tuberculosis, [72]), which are socially transmitted.

A potential shortcoming of our study is that we only had one group of male elk and one group of female elk. It is therefore possible our observed results reflect differences between these groups, as opposed to sex-based differences for elk. Sex-based differences in our results, however, reflect expectations from elk natural history. Thus, we argue our results are unlikely a product of perfect pseudo-replication [73]. For instance, our results at the individual level are similar to those observed for wild elk from at least two systems [13,19]. An additional shortcoming is our sample sizes of individually quantified social network metrics. These were fewer than suggested in a recent power analysis [74] and we acknowledge the need for as many measures as possible for complex mixed generalized linear models such as ours. We are confident, however, our sample size (n = 6 measures per individual) was sufficient to detect biologically relevant within-individual variance in reaction norm intercepts and slopes.

A final caveat of our study is the possibility that repeatability analyses were biased from variation in collar detection range, even though we used Boyland et al.’s [40] correction technique. While proximity collars are commonly used to generate pairwise association matrices, which are, in turn used to construct social networks [75–77], our study is the first to estimate repeatability of social traits derived from proximity collars. The issue of collar bias in repeatability analyses is therefore novel and a standardized solution has not yet been proposed. We suggest two solutions to this problem. First, measuring collar performance and developing a correction for each collar could reduce collar error (for details see Prange et al. [78]). Second, rotating collars among individuals within the same group and re-quantifying repeatability of social traits after rotating collars could also aid in the development of a collar correction metric. Absent the ability to switch collars among the same group of individuals, an alternative would be to deploy proximity collars on new individuals in a different study group or population. In our comparison of social traits derived from the same collars, but deployed on different individuals, we found no correlation, indicating it was unlikely that collar bias influenced our estimates of repeatability (S3 Appendix; S7 Table; S10 Fig). We encourage future studies using social networks derived from proximity collars to further explore this issue.

## Conclusion

Our results suggest that individual social connectedness in captive male and female elk are repeatable, but also display plasticity, across changing local density. Social connectedness meets the criteria for animal personality over time and across an environmental gradient, while both individual male and female elk showed consistent responses to changes in population density, although there was little variation in how individuals responded to changes in density. Our results provide insight into population density as a possible mechanism through which variation in social phenotypes could be maintained in elk. Density dependence is a fundamental process and here we highlight the continued need to revisit our understanding of density dependence as a population-level phenomenon by accounting for consistent individual differences and plasticity and as it pertains to social connectedness [79].

## Supporting information

S1 Appendix Additional information on density replicates. (DOCX)

S2 Appendix Outline for consistency of individual social network position analyses for male and female groups of captive elk. (DOCX)

S3 Appendix Estimating repeatability of social network metrics using proximity collars–a comparison of collar performance across different individual elk. (DOCX)

S4 Appendix Social network matrices generated using proximity data used to construct social networks used for all subsequent analyses. (PDF)

S1 Fig Visual depiction of the experimental enclosures in Saskatchewan (2007) where male and female captive elk (*Cervus canadensis*) herds (females = 12 and males = 11) were contained and moved between to create the three different density treatments (i.e. low, medium and high). (DOCX)

S2 Fig Distribution of mean values of eigenvector centrality (A & D), strength (B & E), and degree (C & F) from randomized networks for female elk (*Cervus canadensis*) at low density (0.71 ha/elk) compared to observed mean values for each metric. Red histograms A-C represent data from ‘replicate 1’ and blue histograms D-F represent data from ‘replicate 2’ (see ‘Methods‘ section for details). Note observed mean values (i.e., vertical thick lines) and 95% quantiles (i.e., vertical dashed lines).
(DOCX)

S3 Fig Distribution of mean values of eigenvector centrality (A & D), strength (B & E), and degree (C & F) from randomized networks for male elk (*Cervus canadensis*) at low density (0.75 ha/elk) compared to observed mean values for each metric. Red histograms A-C represent data from ‘replicate 1’ and blue histograms D-F represent data from ‘replicate 2’ (see ‘Methods‘ section for details). Note observed mean values (i.e., vertical thick lines) and 95% quantiles (i.e., vertical dashed lines).
(DOCX)

S4 Fig Distribution of mean values of eigenvector centrality (A & D), strength (B & E), and degree (C & F) from randomized networks for female elk (*Cervus canadensis*) at medium density (1.05 ha/elk) compared to observed mean values for each metric. Red histograms A-C represent data from ‘replicate 1’ and blue histograms D-F represent data from ‘replicate 2’ (see ‘Methods‘ section for details). Note observed mean values (i.e., vertical thick lines) and 95% quantiles (i.e., vertical dashed lines).
(DOCX)

S5 Fig Distribution of mean values of eigenvector centrality (A & D), strength (B & E), and degree (C & F) from randomized networks for male elk (*Cervus canadensis*) at medium density (1.02 ha/elk) compared to observed mean values for each metric. Red histograms A-C represent data from ‘replicate 1’ and blue histograms D-F represent data from ‘replicate 2’ (see ‘Methods‘ section for details). Note observed mean values (i.e., vertical thick lines) and 95% quantiles (i.e., vertical dashed lines).
(DOCX)

S6 Fig Distribution of mean values of eigenvector centrality (A & D), strength (B & E), and degree (C & F) from randomized networks for female elk (*Cervus canadensis*) at high density (1.43 ha/elk) compared to observed mean values for each metric. Red histograms A-C represent data from ‘replicate 1’ and blue histograms D-F represent data from ‘replicate 2’ (see ‘Methods‘ section for details). Note observed mean values (i.e., vertical thick lines) and 95% quantiles (i.e., vertical dashed lines).
(DOCX)

S7 Fig Distribution of mean values of eigenvector centrality (A & D), strength (B & E), and degree (C & F) from randomized networks for male elk (*Cervus canadensis*) at high density (1.49 ha/elk) compared to observed mean values for each metric. Red histograms A-C represent data from ‘replicate 1’ and blue histograms D-F represent data from ‘replicate 2’ (see ‘Methods‘ section for details). Note observed mean values (i.e., vertical thick lines) and 95% quantiles (i.e., vertical dashed lines).
(DOCX)

S8 Fig Distribution of randomized values of repeatability of three social network metrics for male (A–C) and female (D–F) elk (*Cervus canadensis*). Values of each social network metric were swapped among individuals at each of 1,000 iterations and repeatability was recalculated at each iteration based on the posterior distributions for that model. Vertical red lines represent the observed value of repeatability.
(DOCX)

S9 Fig Variation in social behavior illustrating consistent individual differences (in colour) and population mean (in black) response to changing conspecific density for female (n = 12) and male (n = 11) captive elk in Saskatchewan (2007). Plots illustrate response differences between replicate 1 (left) and replicate 2 (right): (a) and (b) are eigenvector centrality; (c) and (d) are graph strength; and (e) and (f) are degree; for females. For males: (g) and (h) are eigenvector centrality; (I) and (j) are graph strength; and (k) and (l) are degree.
(DOCX)

S10 Fig Relationship between social network metrics calculated based on 13 proximity collars deployed on captive elk (2007) and wild elk in Riding Mountain National Park (2008). Panels A–C represent graph strength calculated from proximity-based social networks for wild (y-axis) and captive (x-axis) generated for each population for groups of elk in high, medium and low density treatments; Panels D–F represent eigenvector centrality calculated from proximity-based social networks for wild (y-axis) and captive (x-axis) generated for each population for groups of elk in high, medium and low density treatments; Panels G–I represent degree calculated from proximity-based social networks for wild (y-axis) and captive (x-axis) generated for each population for groups of elk in high, medium and low density treatments. Note, blue lines represent linear regression, grey shaded area represents standard error around the best-fit line and none of the comparisons were significant (S7 Table).
(DOCX)

S1 Table Summary of hypotheses and predictions for an experimental manipulation of elk (*Cervus canadensis*) herd density (N_Female_ = 12; N_Male_ = 11) to investigate the response of individual social behavior to changing conspecific density. (DOCX)

S2 Table Summary of the randomized order of density treatments for male and female elk (*Cervus canadensis*). Groups of males and females were exposed to each density treatment twice over a six week period. Asterisks refer to treatments where fences from two adjacent small corals were moved to increase the area for a given treatment.
(DOCX)

S3 Table Competing models to explain variation in different measures of individual social centrality (y = eigenvector centrality, graph strength, or degree) of captive male and female elk (*Cervus canadensis*). Models increase in complexity, such that M_1_ is an intercept only null model and M_2_ contains the intercept as a fixed effect and ID as a random intercept to determine if animal personality contributes to variation in sociality. M_3_ contains only density as a fixed effect and M4 –M_6_ contain quadratic responses to density (i.e., density + density^2^) as fixed effects to determine their relationship with sociality, but M_3_ and M_4_ contain only ID as a random intercept. M_5_ and M_6_ are the most complex models containing density and density^2^ as random slopes to determine individual linear and non-linear response to density. Note, asterisks denote models with random effects structures that allow ID to vary as a function of density either linearly (M_5_) or parabolically (M_6_).
(DOCX)

S4 Table Summary of fixed and random effects from the most parsimonious models (DIC < 2) testing effects of density on eigenvector centrality, graph strength and degree in elk (*Cervus canadensis*). (DOCX)

S5 Table Values and ranks for graph strength (panels A and C for males and females, respectively) and eigenvector centrality (panels B and D for males and females, respectively) for each individual elk in our density treatments (low, medium, and high) and replicates (R1 and R2). Note, we did not include raw or ranked data for degree because there was little variation (see Results of main text).
(DOCX)

S6 Table Results of Wilcoxon Rank Sum tests comparing network metric values of replicates 1 and 2 at each density for groups of male and female elk (*Cervus canadensis*) in Saskatchewan (2007). Emboldened values indicate significant differences between metric values of the two replicates.
(DOCX)

S7 Table Summary of Pearson’s correlation coefficients comparing social network metrics derived from collars deployed on captive elk (this study) and wild elk (38). Note, no significant relationship between network metrics calculated in different systems.
(DOCX)
