# Supplementary material for: Consistent individual differences and population plasticity in network-derived sociality: An experimental manipulation of density in a gregarious ungulate
Source: PLoS One. 2018 Mar 1;13(3):e0193425. doi: 10.1371/journal.pone.0193425 (PMC5832262; doi:10.1371/journal.pone.0193425)
Supplement: S1 Appendix — (DOCX) [file pone.0193425.s001.docx]

**Appendix S1.** Additional information on density replicates.

We did not include “replicate” as a fixed or random effect in our models. Given our limited sample size, our models were nearing the point of being over-parameterized with both fixed and random effects. As such, we did not add replicate as an additional covariate. However, we have considered the implications of this issue. To ensure our work is transparent we ran two-sample Wilcoxon rank sum tests with replicates for each sex and each of eigenvector centrality, graph strength, and degree at each of three densities. We found no statistical differences between replicates for all network metrics except female eigenvector centrality, strength and degree at low density and degree at intermediate density (S6 Table). Upon visual examination of the raw metric values plotted against density, it is clear that while there are perceptible differences in metric values between replicates, the overall response to density remains the same (S9 Fig). As such, we decided that it could not be determined whether one replicate was a better representation than another and so replicates were pooled.
