# Supplementary material for: Consistent individual differences and population plasticity in network-derived sociality: An experimental manipulation of density in a gregarious ungulate
Source: PLoS One. 2018 Mar 1;13(3):e0193425. doi: 10.1371/journal.pone.0193425 (PMC5832262; doi:10.1371/journal.pone.0193425)
Supplement: S2 Appendix — (DOCX) [file pone.0193425.s002.docx]

**Appendix S2.** Outline for consistency of individual social network position analyses for male and female groups of captive elk.

We analyzed the consistency of individual social network position across our observed networks following Wilson et al. [1]. In total, we generated six values of degree, strength and eigenvector centrality per individual: two replicates at each of three density treatments. We only conducted these randomizations for graph strength and eigenvector centrality because there was little variation in degree, which would result in all individuals being tied during the ranking process. We ranked network metrics within each replicate by treatment combination (for raw data see S3 Table) and assessed the relative change in an individual’s network position compared to other individuals in the network. The test developed by Wilson et al. [1] randomizes the ranks of individual network metrics and compares the sum of individual variances of observed ranks across all networks to the sum of individual variances from randomly generated networks. This test assumes the probability of observation is equal for all individuals, which was the case for elk in our study system. Significance was determined by comparing the observed sum of variances to a distribution of random sum of variances generated from 10,000 randomizations of observed data. To perform randomizations we used code provided by Krause et al. [2] which is available at <https://cosa.fh-luebeck.de/download/skrause/>.

When considering graph strength, we found that individual network position for males (p = 0.001) and females (p = 0.0001) was consistent across all networks. Meanwhile, for centrality, we found that individual network position for males (p = 0.02) and females (p = 0.0001) was consistent across all networks.

**References**

1. Wilson ADM, Krause S, Dingemanse NJ, Krause J. Network position: A key component in the characterization of social personality types. Behav Ecol Sociobiol. 2013;67(1):163–73.

2. Krause S, Wilson ADM, Ramnarine IW, Herbert-Read JE, Clément RJG, Krause J. Guppies occupy consistent positions in social networks: mechanisms and consequences. Behav Ecol. 2017;28:429–38.
