# Supplementary material for: Consistent individual differences and population plasticity in network-derived sociality: An experimental manipulation of density in a gregarious ungulate
Source: PLoS One. 2018 Mar 1;13(3):e0193425. doi: 10.1371/journal.pone.0193425 (PMC5832262; doi:10.1371/journal.pone.0193425)
Supplement: S3 Appendix — (DOCX) [file pone.0193425.s003.docx]

**Appendix S3.** Estimating repeatability of social network metrics using proximity collars – a comparison of collar performance across different elk.

To assess whether bias associated with proximity collars (described by Boyland et al. [1]

influenced our measures of repeatability we deployed the same collars (Sirtrack Proximity Logger radio-collars; Sirtrack Ltd., Havelock North, NZ) on free-ranging elk in Riding Mountain National Park (RMNP: Vander Wal et al. [2]). Collars were originally deployed on elk in this study in 2007 and the same collars were subsequently deployed on elk in western RMNP in 2008. Elk in RMNP are separated into two distinct sub-populations, one in eastern and one in western RMNP and there is no overlap between these sub-populations [3,4]. In total, 23 proximity collars were deployed on elk in western RMNP in 2008 [2] using a net-gun fired from a helicopter [5]. Of the 23 collars deployed in western RMNP [2,3], 13 were also deployed on elk used in this study.

We used the same social network procedures described in this study to generate social networks for elk in RMNP (Webber and Vander Wal, unpublished data). For each of the 13 collars used in both study systems, we calculated graph strength, eigenvector centrality, and degree and assessed the correlation between metrics generated based on collars deployed on elk in captive vs. wild systems (S7 Table). We found no relationship between graph strength generated for collars between systems, suggesting that, at least based on our conservative test, proximity collars likely had little effect on repeatability of social network metrics for captive elk.

**References**

1. Boyland NK, James R, Mlynski DT, Madden JR, Croft DP. Spatial proximity loggers for recording animal social networks: Consequences of inter-logger variation in performance. Behav Ecol Sociobiol. 2013;67(11):1877–90.

2. Vander Wal E, Paquet PC, Messier F, Mcloughlin PD. Effects of phenology and sex on social proximity in a gregarious ungulate. Can J Zool. 2013;91:601–9.

3. Vander Wal E, Paquet PC, Andraés J a. Influence of landscape and social interactions on transmission of disease in a social cervid. Mol Ecol. 2012;21(5):1271–82.

4. Vander Wal E, Laforge MP, McLoughlin PD. Density dependence in social behaviour: home range overlap and density interacts to affect conspecific encounter rates in a gregarious ungulate. Behav Ecol Sociobiol. 2014;68(3):383–90.

5. Cattet MRL, Caulkett N a, Wilson C, Vandenbrink T, Brook RK. Intranasal administration of xylazine to reduce stress in elk captured by net gun. J Wildl Dis. 2004;40(3):562–5.
