# Supplementary material for: Consistent individual differences and population plasticity in network-derived sociality: An experimental manipulation of density in a gregarious ungulate
Source: PLoS One. 2018 Mar 1;13(3):e0193425. doi: 10.1371/journal.pone.0193425 (PMC5832262; doi:10.1371/journal.pone.0193425)
Supplement: S4 Appendix — (PDF) [file pone.0193425.s004.pdf]

Appendix S4. Social network matrices generated using proximity data used to construct social networks used for all subsequent analyses.

Meta-data file naming scheme:

m = matrix

F = female; M = male

t = treatment

H = high; M = Medium; L = low (see main text for specific treatment densities)

r = replicate

1 = replicate 1; 2 = replicate 2

	c26	c27	c28	c29	c30	c31	c33	c34	c35	c37	c39	c69
c26	0.00	8.50	7.85	4.17	15.53	37.05	4.34	2.09	0.59	16.83	2.00	12.18
c27	0.00	0.00	10.27	8.56	2.84	4.71	8.00	9.37	0.64	5.32	3.49	4.28
c28	0.00	0.00	0.00	8.71	4.67	10.87	12.73	12.92	1.00	21.22	2.92	4.67
c29	0.00	0.00	0.00	0.00	3.21	8.25	7.97	9.80	0.86	14.33	1.68	9.96
c30	0.00	0.00	0.00	0.00	0.00	21.48	2.20	1.38	0.00	4.89	1.25	2.80
c31	0.00	0.00	0.00	0.00	0.00	0.00	9.65	5.47	1.43	17.07	4.98	11.76
c33	0.00	0.00	0.00	0.00	0.00	0.00	0.00	14.70	0.80	11.82	6.80	5.90
c34	0.00	0.00	0.00	0.00	0.00	0.00	0.00	0.00	0.88	7.44	5.75	3.05
c35	0.00	0.00	0.00	0.00	0.00	0.00	0.00	0.00	0.00	2.67	0.75	1.50
c37	0.00	0.00	0.00	0.00	0.00	0.00	0.00	0.00	0.00	0.00	8.81	8.82
c39	0.00	0.00	0.00	0.00	0.00	0.00	0.00	0.00	0.00	0.00	0.00	6.20
c69	0.00	0.00	0.00	0.00	0.00	0.00	0.00	0.00	0.00	0.00	0.00	0.00

mFtHr2

	c26	c27	c28	c29	c30	c31	c33	c34	c35	c37	c39	c69
c26	0.000	2.429	3.857	3.937	5.275	14.226	4.018	5.106	1.984	3.214	0.462	13.617
c27	0.000	0.000	4.416	4.126	1.734	3.646	4.966	6.040	1.535	2.636	2.210	6.789
c28	0.000	0.000	0.000	5.913	4.484	5.271	7.696	17.217	1.238	7.286	2.989	6.586
c29	0.000	0.000	0.000	0.000	1.389	16.620	8.785	6.101	1.111	12.222	1.632	11.626
c30	0.000	0.000	0.000	0.000	0.000	9.870	1.399	2.749	1.462	6.231	0.923	3.684
c31	0.000	0.000	0.000	0.000	0.000	0.000	4.766	4.128	0.889	7.921	1.077	16.947
c33	0.000	0.000	0.000	0.000	0.000	0.000	0.000	10.166	3.451	5.938	2.620	10.125
c34	0.000	0.000	0.000	0.000	0.000	0.000	0.000	0.000	1.860	7.435	3.472	4.989
c35	0.000	0.000	0.000	0.000	0.000	0.000	0.000	0.000	0.000	1.389	0.419	1.439
c37	0.000	0.000	0.000	0.000	0.000	0.000	0.000	0.000	0.000	0.000	5.385	7.921
c39	0.000	0.000	0.000	0.000	0.000	0.000	0.000	0.000	0.000	0.000	0.000	0.984
c69	0.000	0.000	0.000	0.000	0.000	0.000	0.000	0.000	0.000	0.000	0.000	0.000

mFtLr1

	c26	c27	c28	c29	c30	c31	c33	c34	c35	c37	c39	c69
c26	0.000	5.986	7.783	2.936	31.791	46.980	20.657	10.238	6.359	13.672	4.154	18.446
c27	0.000	0.000	14.957	6.228	6.163	5.743	17.827	15.108	2.237	4.243	4.457	13.304
c28	0.000	0.000	0.000	11.698	13.176	12.500	16.360	14.933	1.124	14.400	4.029	11.344
c29	0.000	0.000	0.000	0.000	3.939	8.416	22.663	13.217	3.765	16.186	3.846	16.223
c30	0.000	0.000	0.000	0.000	0.000	29.421	12.035	6.294	2.614	15.703	3.978	8.926
c31	0.000	0.000	0.000	0.000	0.000	0.000	11.156	7.478	3.189	22.857	6.914	13.558
c33	0.000	0.000	0.000	0.000	0.000	0.000	0.000	25.300	5.844	19.465	6.570	19.213
c34	0.000	0.000	0.000	0.000	0.000	0.000	0.000	0.000	4.652	9.411	4.175	10.618
c35	0.000	0.000	0.000	0.000	0.000	0.000	0.000	0.000	0.000	5.529	0.975	3.178
c37	0.000	0.000	0.000	0.000	0.000	0.000	0.000	0.000	0.000	0.000	7.971	21.747
c39	0.000	0.000	0.000	0.000	0.000	0.000	0.000	0.000	0.000	0.000	0.000	6.862
c69	0.000	0.000	0.000	0.000	0.000	0.000	0.000	0.000	0.000	0.000	0.000	0.000

mFtLr2

	c26	c27	c28	c29	c30	c31	c33	c34	c35	c37	c39	c69
c26	0.000	1.000	7.500	2.833	5.000	29.500	5.833	2.000	0.500	3.500	0.500	6.500
c27	0.000	0.000	1.000	2.667	1.000	2.000	19.667	7.000	1.000	1.667	1.000	6.500
c28	0.000	0.000	0.000	20.333	2.000	3.000	17.667	11.000	1.000	8.833	0.500	3.500
c29	0.000	0.000	0.000	0.000	2.333	50.667	14.667	4.000	2.333	9.333	0.333	3.667
c30	0.000	0.000	0.000	0.000	0.000	13.000	2.333	0.000	0.000	3.333	0.000	0.000
c31	0.000	0.000	0.000	0.000	0.000	0.000	10.333	1.500	1.500	7.000	0.500	8.000
c33	0.000	0.000	0.000	0.000	0.000	0.000	0.000	9.667	0.667	11.000	0.000	3.667
c34	0.000	0.000	0.000	0.000	0.000	0.000	0.000	0.000	0.000	4.667	0.000	0.000
c35	0.000	0.000	0.000	0.000	0.000	0.000	0.000	0.000	0.000	1.333	0.000	0.000
c37	0.000	0.000	0.000	0.000	0.000	0.000	0.000	0.000	0.000	0.000	4.000	6.667
c39	0.000	0.000	0.000	0.000	0.000	0.000	0.000	0.000	0.000	0.000	0.000	0.000
c69	0.000	0.000	0.000	0.000	0.000	0.000	0.000	0.000	0.000	0.000	0.000	0.000

mFtMr1

	c26	c27	c28	c29	c30	c31	c33	c34	c35	c37	c39	c69
c26	0.000	13.448	11.233	8.209	39.949	61.111	22.410	13.633	7.522	13.015	0.786	23.841
c27	0.000	0.000	26.703	25.290	11.311	19.914	31.351	29.854	6.403	31.291	4.591	18.633
c28	0.000	0.000	0.000	22.022	5.200	14.300	18.500	29.400	5.058	18.746	2.357	15.232
c29	0.000	0.000	0.000	0.000	7.778	19.222	31.444	23.433	4.267	44.286	3.984	23.670
c30	0.000	0.000	0.000	0.000	0.000	36.944	11.000	2.600	7.839	22.256	1.429	7.839
c31	0.000	0.000	0.000	0.000	0.000	0.000	18.028	18.372	7.425	25.953	2.071	21.482
c33	0.000	0.000	0.000	0.000	0.000	0.000	0.000	26.700	8.306	34.524	3.143	30.000
c34	0.000	0.000	0.000	0.000	0.000	0.000	0.000	0.000	11.845	27.205	6.329	10.948
c35	0.000	0.000	0.000	0.000	0.000	0.000	0.000	0.000	0.000	6.832	2.090	4.645
c37	0.000	0.000	0.000	0.000	0.000	0.000	0.000	0.000	0.000	0.000	5.620	24.747
c39	0.000	0.000	0.000	0.000	0.000	0.000	0.000	0.000	0.000	0.000	0.000	5.433
c69	0.000	0.000	0.000	0.000	0.000	0.000	0.000	0.000	0.000	0.000	0.000	0.000

mFtMr2

	c26	c27	c28	c29	c30	c31	c33	c34	c35	c37	c39	c69
c26	0.000	0.500	8.000	4.500	38.000	71.167	17.000	16.500	19.500	16.000	0.000	25.800
c27	0.000	0.000	1.000	0.000	0.500	3.333	4.375	5.000	0.000	2.000	0.000	2.200
c28	0.000	0.000	0.000	24.500	7.000	15.333	45.125	42.000	12.000	28.500	3.500	26.000
c29	0.000	0.000	0.000	0.000	5.500	53.500	49.000	30.500	12.500	27.500	4.500	24.600
c30	0.000	0.000	0.000	0.000	0.000	32.833	16.500	2.500	14.000	29.000	1.000	7.400
c31	0.000	0.000	0.000	0.000	0.000	0.000	20.000	21.333	12.000	28.000	3.333	37.033
c33	0.000	0.000	0.000	0.000	0.000	0.000	0.000	35.625	29.625	47.750	5.000	26.675
c34	0.000	0.000	0.000	0.000	0.000	0.000	0.000	0.000	12.500	32.000	5.000	11.600
c35	0.000	0.000	0.000	0.000	0.000	0.000	0.000	0.000	0.000	29.500	1.500	10.900
c37	0.000	0.000	0.000	0.000	0.000	0.000	0.000	0.000	0.000	0.000	3.500	24.400
c39	0.000	0.000	0.000	0.000	0.000	0.000	0.000	0.000	0.000	0.000	0.000	7.200
c69	0.000	0.000	0.000	0.000	0.000	0.000	0.000	0.000	0.000	0.000	0.000	0.000

mMtHr1

	c14	c19	c24	c40	c49	c8	c80	c81	c82	c83	c84
c14	0.000	0.500	26.500	1.358	31.500	42.500	31.500	26.500	9.000	63.000	61.000
c19	0.000	0.000	0.000	82.209	0.000	0.000	0.000	1.000	0.000	0.500	0.500
c24	0.000	0.000	0.000	1.216	14.000	25.500	20.000	10.500	5.000	24.500	54.000
c40	0.000	0.000	0.000	0.000	0.000	0.358	0.358	0.000	0.858	1.358	1.358
c49	0.000	0.000	0.000	0.000	0.000	30.000	21.000	14.500	11.500	41.000	57.500
c8	0.000	0.000	0.000	0.000	0.000	0.000	23.000	28.500	4.000	37.500	83.000
c80	0.000	0.000	0.000	0.000	0.000	0.000	0.000	19.500	6.500	30.000	46.000
c81	0.000	0.000	0.000	0.000	0.000	0.000	0.000	0.000	5.000	81.000	36.000
c82	0.000	0.000	0.000	0.000	0.000	0.000	0.000	0.000	0.000	39.500	16.000
c83	0.000	0.000	0.000	0.000	0.000	0.000	0.000	0.000	0.000	0.000	125.000
c84	0.000	0.000	0.000	0.000	0.000	0.000	0.000	0.000	0.000	0.000	0.000

mMtHr2

	c14	c19	c24	c40	c49	c8	c80	c81	c82	c83	c84
c14	0.000	0.000	13.151	0.000	16.959	34.041	21.818	12.508	4.400	18.474	38.914
c19	0.000	0.000	0.467	72.000	0.819	0.340	0.000	0.268	0.798	0.000	1.045
c24	0.000	0.000	0.000	0.500	11.873	17.020	15.467	16.448	3.783	8.867	25.373
c40	0.000	0.000	0.000	0.000	0.819	0.500	0.817	0.768	0.000	0.000	0.500
c49	0.000	0.000	0.000	0.000	0.000	27.136	15.584	11.167	7.340	17.713	35.472
c8	0.000	0.000	0.000	0.000	0.000	0.000	25.987	18.035	3.361	19.720	63.405
c80	0.000	0.000	0.000	0.000	0.000	0.000	0.000	25.992	9.478	22.483	43.386
c81	0.000	0.000	0.000	0.000	0.000	0.000	0.000	0.000	4.319	50.513	31.619
c82	0.000	0.000	0.000	0.000	0.000	0.000	0.000	0.000	0.000	16.830	8.685
c83	0.000	0.000	0.000	0.000	0.000	0.000	0.000	0.000	0.000	0.000	63.000
c84	0.000	0.000	0.000	0.000	0.000	0.000	0.000	0.000	0.000	0.000	0.000

MMtLr1

	c14	c19	c24	c40	c49	c8	c80	c81	c82	c83	c84
c14	0.000	2.200	4.857	2.000	3.333	6.000	7.077	2.000	0.000	5.000	3.929
c19	0.000	0.000	0.000	89.700	0.000	0.400	0.000	0.000	0.000	0.000	0.714
c24	0.000	0.000	0.000	0.500	3.905	2.714	6.593	2.571	1.143	4.476	12.143
c40	0.000	0.000	0.000	0.000	0.000	3.500	0.000	0.000	0.000	0.000	1.214
c49	0.000	0.000	0.000	0.000	0.000	17.167	8.256	0.000	1.333	10.000	13.000
c8	0.000	0.000	0.000	0.000	0.000	0.000	9.154	3.000	0.500	6.500	13.214
c80	0.000	0.000	0.000	0.000	0.000	0.000	0.000	10.423	0.615	4.462	9.527
c81	0.000	0.000	0.000	0.000	0.000	0.000	0.000	0.000	0.000	29.833	2.000
c82	0.000	0.000	0.000	0.000	0.000	0.000	0.000	0.000	0.000	2.000	1.429
c83	0.000	0.000	0.000	0.000	0.000	0.000	0.000	0.000	0.000	0.000	40.238
c84	0.000	0.000	0.000	0.000	0.000	0.000	0.000	0.000	0.000	0.000	0.000

mMtLr2

	c14	c19	c24	c40	c49	c8	c80	c81	c82	c83	c84
c14	0.000	0.000	2.636	0.000	3.375	5.227	4.000	1.167	0.000	3.333	8.433
c19	0.000	0.000	0.000	40.500	1.000	0.000	0.000	0.000	0.000	0.500	0.000
c24	0.000	0.000	0.000	0.000	1.864	3.091	9.682	6.182	0.727	1.924	15.170
c40	0.000	0.000	0.000	0.000	0.875	0.000	0.000	2.000	1.500	1.333	0.500
c49	0.000	0.000	0.000	0.000	0.000	13.943	8.250	7.875	3.750	16.292	26.633
c8	0.000	0.000	0.000	0.000	0.000	0.000	21.136	11.333	0.455	5.227	67.885
c80	0.000	0.000	0.000	0.000	0.000	0.000	0.000	16.333	1.500	6.167	41.767
c81	0.000	0.000	0.000	0.000	0.000	0.000	0.000	0.000	1.333	25.000	29.200
c82	0.000	0.000	0.000	0.000	0.000	0.000	0.000	0.000	0.000	5.000	6.600
c83	0.000	0.000	0.000	0.000	0.000	0.000	0.000	0.000	0.000	0.000	35.400
c84	0.000	0.000	0.000	0.000	0.000	0.000	0.000	0.000	0.000	0.000	0.000

mMtMr1

	c14	c19	c24	c40	c49	c8	c80	c81	c82	c83	c84
c14	0.000	0.000	8.167	0.000	2.556	13.125	8.750	2.714	2.667	8.433	18.235
c19	0.000	0.000	0.333	82.500	0.722	1.125	0.000	0.286	0.500	0.000	0.000
c24	0.000	0.000	0.000	0.333	3.889	8.833	6.667	3.762	3.500	13.667	22.902
c40	0.000	0.000	0.000	0.000	1.444	0.000	0.000	0.000	1.167	0.500	2.676
c49	0.000	0.000	0.000	0.000	0.000	8.514	7.611	3.286	3.056	11.778	17.412
c8	0.000	0.000	0.000	0.000	0.000	0.000	14.125	6.946	2.083	26.108	47.353
c80	0.000	0.000	0.000	0.000	0.000	0.000	0.000	6.250	1.917	10.867	17.059
c81	0.000	0.000	0.000	0.000	0.000	0.000	0.000	0.000	1.429	28.190	8.832
c82	0.000	0.000	0.000	0.000	0.000	0.000	0.000	0.000	0.000	10.367	10.549
c83	0.000	0.000	0.000	0.000	0.000	0.000	0.000	0.000	0.000	0.000	50.655
c84	0.000	0.000	0.000	0.000	0.000	0.000	0.000	0.000	0.000	0.000	0.000

mMtMr2

	c14	c19	c24	c40	c49	c8	c80	c81	c82	c83	c84
c14	0.000	0.000	3.214	0.000	3.333	1.167	1.000	2.429	1.000	2.500	7.077
c19	0.000	0.000	2.143	97.500	0.000	1.667	1.000	0.857	0.500	1.000	1.115
c24	0.000	0.000	0.000	0.429	2.548	3.571	9.143	3.429	1.429	2.286	16.121
c40	0.000	0.000	0.000	0.000	2.000	0.000	0.000	0.286	0.000	1.000	3.231
c49	0.000	0.000	0.000	0.000	0.000	10.667	15.500	7.881	1.833	6.167	11.013
c8	0.000	0.000	0.000	0.000	0.000	0.000	15.667	2.905	0.500	4.667	29.231
c80	0.000	0.000	0.000	0.000	0.000	0.000	0.000	14.429	3.500	10.000	34.154
c81	0.000	0.000	0.000	0.000	0.000	0.000	0.000	0.000	1.571	9.143	21.077
c82	0.000	0.000	0.000	0.000	0.000	0.000	0.000	0.000	0.000	18.500	4.962
c83	0.000	0.000	0.000	0.000	0.000	0.000	0.000	0.000	0.000	0.000	21.231
c84	0.000	0.000	0.000	0.000	0.000	0.000	0.000	0.000	0.000	0.000	0.000
