# Supplementary material for: Consistent individual differences and population plasticity in network-derived sociality: An experimental manipulation of density in a gregarious ungulate
Source: PLoS One. 2018 Mar 1;13(3):e0193425. doi: 10.1371/journal.pone.0193425 (PMC5832262; doi:10.1371/journal.pone.0193425)
Supplement: S1 Fig — (DOCX) [file pone.0193425.s005.docx]

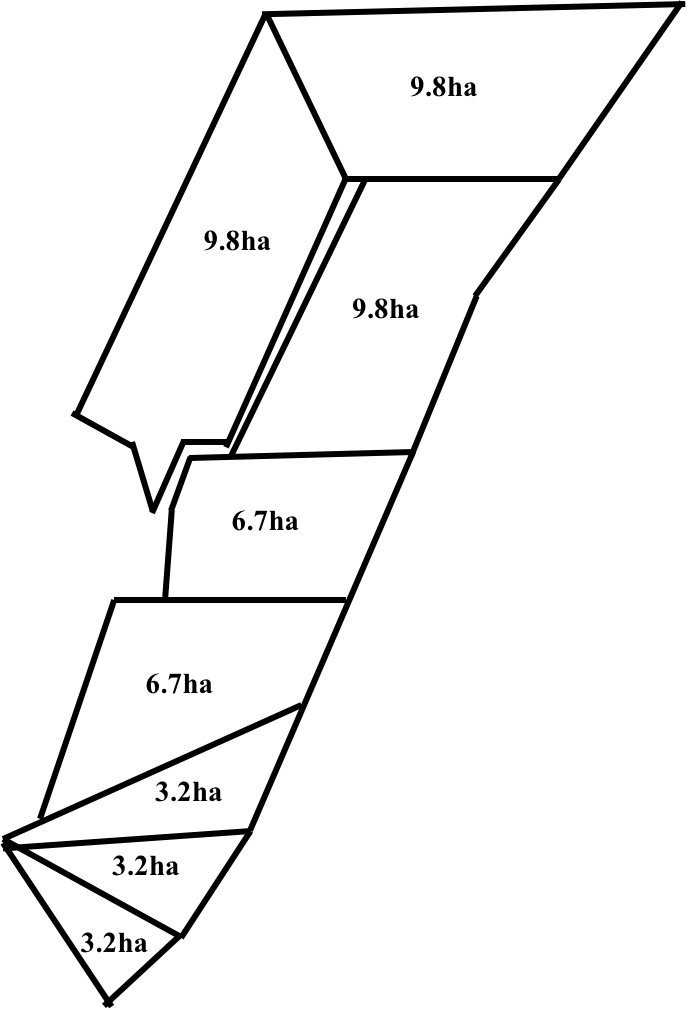


**Fig S1.** Visual depiction of the experimental enclosures in Saskatchewan (2007) where male and female captive elk (*Cervus canadensis*) herds (females = 12 and males = 11) were contained and moved between to create the three different density treatments (i.e. low, medium and high).
