# Supplementary material for: Consistent individual differences and population plasticity in network-derived sociality: An experimental manipulation of density in a gregarious ungulate
Source: PLoS One. 2018 Mar 1;13(3):e0193425. doi: 10.1371/journal.pone.0193425 (PMC5832262; doi:10.1371/journal.pone.0193425)
Supplement: S5 Fig — Distribution of mean values of eigenvector centrality (A & D), strength (B & E), and degree (C & F) from randomized networks for male elk (Cervus canadensis) at medium density (1.02 ha/elk) compared to observed mean values for each metric. Red histograms A-C represent data from ‘replicate 1’ and blue histograms D-F represent data from ‘replicate 2’ (see ‘Methods‘ section for details). Note observed mean values (i.e., vertical thick lines) and 95% quantiles (i.e., vertical dashed lines). (DOCX) [file pone.0193425.s009.docx]

**Fig S5.** Distribution of mean values of eigenvector centrality (A & D), strength (B & E), and degree (C & F) from randomized networks for male elk (*Cervus canadensis*) at medium density (1.02 ha/elk) compared to observed mean values for each metric. Red histograms A-C represent data from ‘replicate 1’ and blue histograms D-F represent data from ‘replicate 2’ (see ‘Methods’ section for details). Note observed mean values (i.e., vertical thick lines) and 95% quantiles (i.e., vertical dashed lines).
