# Supplementary material for: Consistent individual differences and population plasticity in network-derived sociality: An experimental manipulation of density in a gregarious ungulate
Source: PLoS One. 2018 Mar 1;13(3):e0193425. doi: 10.1371/journal.pone.0193425 (PMC5832262; doi:10.1371/journal.pone.0193425)
Supplement: S8 Fig — Distribution of randomized values of repeatability of three social network metrics for male (A–C) and female (D–F) elk (Cervus canadensis). Values of each social network metric were swapped among individuals at each of 1,000 iterations and repeatability was recalculated at each iteration based on the posterior distributions for that model. Vertical red lines represent the observed value of repeatability. (DOCX) [file pone.0193425.s012.docx]

**Fig S8.** Distribution of randomized values of repeatability of three social network metrics for male (A–C) and female (D–F) elk (*Cervus canadensis*). Values of each social network metric were swapped among individuals at each of 1,000 iterations and repeatability was recalculated at each iteration based on the posterior distributions for that model. Vertical red lines represent the observed value of repeatability.
