# Supplementary material for: Consistent individual differences and population plasticity in network-derived sociality: An experimental manipulation of density in a gregarious ungulate
Source: PLoS One. 2018 Mar 1;13(3):e0193425. doi: 10.1371/journal.pone.0193425 (PMC5832262; doi:10.1371/journal.pone.0193425)
Supplement: S9 Fig — Plots illustrate response differences between replicate 1 (left) and replicate 2 (right): (a) and (b) are eigenvector centrality; (c) and (d) are graph strength; and (e) and (f) are degree; for females. For males: (g) and (h) are eigenvector centrality; (I) and (j) are graph strength; and (k) and (l) are degree. (DOCX) [file pone.0193425.s013.docx]

**
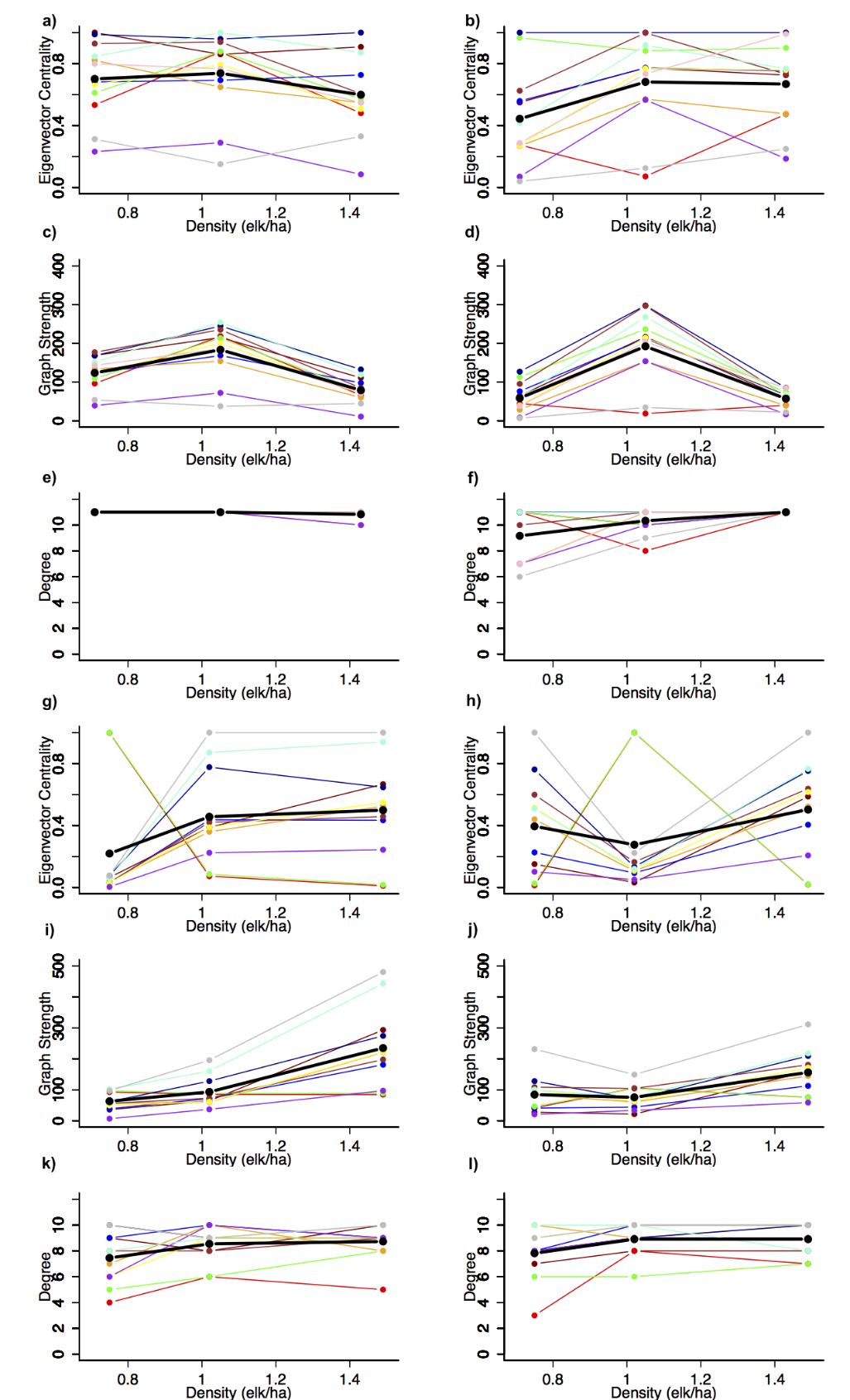
**

**Fig S9.** Variation in social behavior illustrating consistent individual differences (in colour) and population mean (in black) response to changing conspecific density for female (n = 12) and male (n = 11) captive elk in Saskatchewan (2007). Plots illustrate response differences between replicate 1 (left) and replicate 2 (right): (a) and (b) are eigenvector centrality; (c) and (d) are graph strength; and (e) and (f) are degree; for females. For males: (g) and (h) are eigenvector centrality; (I) and (j) are graph strength; and (k) and (l) are degree.
