# Supplementary material for: Consistent individual differences and population plasticity in network-derived sociality: An experimental manipulation of density in a gregarious ungulate
Source: PLoS One. 2018 Mar 1;13(3):e0193425. doi: 10.1371/journal.pone.0193425 (PMC5832262; doi:10.1371/journal.pone.0193425)
Supplement: S10 Fig — Panels A–C represent graph strength calculated from proximity-based social networks for wild (y-axis) and captive (x-axis) generated for each population for groups of elk in high, medium and low density treatments; Panels D–F represent eigenvector centrality calculated from proximity-based social networks for wild (y-axis) and captive (x-axis) generated for each population for groups of elk in high, medium and low density treatments; Panels G–I represent degree calculated from proximity-based social networks for wild (y-axis) and captive (x-axis) generated for each population for groups of elk in high, medium and low density treatments. Note, blue lines represent linear regression, grey shaded area represents standard error around the best-fit line and none of the comparisons were significant (S7 Table). (DOCX) [file pone.0193425.s014.docx]

**Fig S10.** Relationship between social network metrics calculated based on 13 proximity collars deployed on captive elk (2007) and wild elk in Riding Mountain National Park (2008). Panels A – C represent graph strength calculated from proximity-based social networks for wild (y-axis) and captive (x-axis) generated for each population for groups of elk in high, medium and low density treatments; Panels D – F represent eigenvector centrality calculated from proximity-based social networks for wild (y-axis) and captive (x-axis) generated for each population for groups of elk in high, medium and low density treatments; Panels G – I represent degree calculated from proximity-based social networks for wild (y-axis) and captive (x-axis) generated for each population for groups of elk in high, medium and low density treatments. Note, black trend lines represent best-fit linear regressions; none of the comparisons were significant (Table S7).
