# Supplementary material for: Consistent individual differences and population plasticity in network-derived sociality: An experimental manipulation of density in a gregarious ungulate
Source: PLoS One. 2018 Mar 1;13(3):e0193425. doi: 10.1371/journal.pone.0193425 (PMC5832262; doi:10.1371/journal.pone.0193425)
Supplement: S1 Table — (DOCX) [file pone.0193425.s015.docx]

**Table S1.** Summary of hypotheses and predictions for an experimental manipulation of elk (*Cervus canadensis*) herd density (N_Female_ = 12; N_Male_ = 11) to investigate the response of individual social behavior to changing conspecific density.

| Hypotheses | Predictions |
| --- | --- |
| 1. Among-individual social connectedness will increase with increasing density. | a) Female elk, given that they form groups in the non-breeding season to reduce vigilance and increase foraging, and have been shown to have highest frequency and duration of interactions at intermediate density (8), would exhibit highest centrality at an intermediate density. |
|  | b) Male elk, given that vigilance is primarily directed towards conspecifics, and interaction frequency increases while duration decreases with increasing density (8), would exhibit increased centrality with increasing density. |
| 2. Within-individual social connectedness will be repeatable and plastic across densities. | a) If repeatable, individuals will exhibit within-individual differences in reaction norm intercepts (i.e., individuals who are more central, will always be more central regardless of density). |
|  | b) If plastic, individuals will exhibit within-individual differences in reaction norm slopes. |
