# Supplementary material for: Consistent individual differences and population plasticity in network-derived sociality: An experimental manipulation of density in a gregarious ungulate
Source: PLoS One. 2018 Mar 1;13(3):e0193425. doi: 10.1371/journal.pone.0193425 (PMC5832262; doi:10.1371/journal.pone.0193425)
Supplement: S2 Table — Groups of males and females were exposed to each density treatment twice over a six week period. Asterisks refer to treatments where fences from two adjacent small corals were moved to increase the area for a given treatment. (DOCX) [file pone.0193425.s016.docx]

**Table S2.** Summary of the randomized order of density treatments for male and female elk (*Cervus canadensis*). Groups of males and females were exposed to each density treatment twice over a six week period. Asterisks refer to treatments where fences from two adjacent small corals were moved to increase the area for a given treatment.

|  |  | **Males** | | | **Females** | | |
| --- | --- | --- | --- | --- | --- | --- | --- |
| **Week** | **Replicate** | **Density (ha)** | **Treatment** | **Number of Encounters** | **Density (ha)** | **Treatment** | **Number of Encounters** |
| 1 | 1 | 6.7 | High | 1292.57 | 19.6 (9.8 + 9.8)* | Low | 742.72 |
| 2 | 1 | 13.4  (6.7 + 6.7)* | Low | 348.62 | 9.8 | High | 474.60 |
| 3 | 1 | 9.8 | Medium | 513.84 | 13.4 | Medium | 1101.01 |
| 4 | 2 | 6.7 | High | 859.96 | 9.8 | High | 344.02 |
| 5 | 2 | 13.4  (6.7 + 6.7)* | Low | 466.10 | 19.6 (9.8 + 9.8)* | Low | 353.50 |
| 6 | 2 | 9.8 | Medium | 417.57 | 13.4 | Medium | 1154.64 |
