# Supplementary material for: Consistent individual differences and population plasticity in network-derived sociality: An experimental manipulation of density in a gregarious ungulate
Source: PLoS One. 2018 Mar 1;13(3):e0193425. doi: 10.1371/journal.pone.0193425 (PMC5832262; doi:10.1371/journal.pone.0193425)
Supplement: S3 Table — Models increase in complexity, such that M1 is an intercept only null model and M2 contains the intercept as a fixed effect and ID as a random intercept to determine if animal personality contributes to variation in sociality. M3 contains only density as a fixed effect and M4 –M6 contain quadratic responses to density (i.e., density + density2) as fixed effects to determine their relationship with sociality, but M3 and M4 contain only ID as a random intercept. M5 and M6 are the most complex models containing density and density2 as random slopes to determine individual linear and non-linear response to density. Note, asterisks denote models with random effects structures that allow ID to vary as a function of density either linearly (M5) or parabolically (M6). (DOCX) [file pone.0193425.s017.docx]

**Table S3.** Competing models to explain variation in different measures of individual social centrality (y = eigenvector centrality, graph strength, or degree) of captive male and female elk (*Cervus canadensis*). Models increase in complexity, such that M_1_ is an intercept only null model and M_2_ contains the intercept as a fixed effect and ID as a random intercept to determine if animal personality contributes to variation in sociality. M_3_ contains only density as a fixed effect and M_4_ –M_6_ contain quadratic responses to density (i.e., density + density^2^) as fixed effects to determine their relationship with sociality, but M_3_ and M_4_ contain only ID as a random intercept. M_5_ and M_6_ are the most complex models containing density and density^2^ as random slopes to determine individual linear and non-linear response to density. Note, asterisks denote models with random effects structures that allow ID to vary as a function of density either linearly (M_5_) or parabolically (M_6_).

| **Model** | **Equation** |
| --- | --- |
| M_1_ | y ~ 1 |
| M_2_ | y ~ 1 + (1\|ID) |
| M_3_ | y ~ Density + (1\|ID) |
| M_4_ | y ~ Density + Density^2^ + (1\|ID) |
| M_5_* | y ~ Density + Density^2^ + (1\|ID) + (1\|Density):ID |
| M_6_* | y ~ Density + Density^2^ + (1\|ID) + (1\|Density):ID + (1\|Density^2^):ID |
