# Supplementary material for: Consistent individual differences and population plasticity in network-derived sociality: An experimental manipulation of density in a gregarious ungulate
Source: PLoS One. 2018 Mar 1;13(3):e0193425. doi: 10.1371/journal.pone.0193425 (PMC5832262; doi:10.1371/journal.pone.0193425)
Supplement: S4 Table — (DOCX) [file pone.0193425.s018.docx]

**Table S4.** Summary of fixed and random effects from the most parsimonious models (DIC < 2) testing effects of density on eigenvector centrality, graph strength and degree in elk (*Cervus canadensis*).

|  | **Fixed effects** | | | | **Random effects** | | |
| --- | --- | --- | --- | --- | --- | --- | --- |
| **Males** | **Coefficient** | **2.5% CI** | **97.5% CI** | **P-value** | **Variance** | **2.5% CI** | **97.5% CI** |
| **Centrality** |  |  |  |  |  |  |  |
| -Density | 0.032 | -2.71 | 3.0 | 0.98 | - | - | - |
| -Density^2^ | 0.10 | -1.06 | 1.47 | 0.89 | - | - | - |
| -ID | - | - | - | - | 0.031 | 0.005 | 0.077 |
| -Density | - | - | - | - | 0.031 | 0.005 | 0.086 |
| -Density^2^ | - | - | - | - | 0.026 | 0.005 | 0.062 |
| -Residual | - | - | - | - | 0.099 | 0.062 | 0.138 |
| **Strength** |  |  |  |  |  |  |  |
| -Density | -426.42 | -851.92 | 30.40 | 0.06 | - | - | - |
| -Density^2^ | 264.52 | 78.31 | 467.12 | 0.01 | - | - | - |
| -ID | - | - | - | - | 1,728 | 357.6 | 4,192 |
| -Density | - | - | - | - | 2,198 | 243.4 | 5,561 |
| -Density^2^ | - | - | - | - | 2,344 | 481.3 | 5,389 |
| -Residual | - | - | - | - | 2,278 | 1,427 | 5,389 |
| **Degree** |  |  |  |  |  |  |  |
| -Density | 13.21 | 3.85 | 6.03 | 0.80 | - | - | - |
| -Density^2^ | -5.18 | -9.16 | -0.45 | 0.01 | - | - | - |
| -ID | - | - | - | - | 1.77 | 0.31 | 4.30 |
| -Density | - | - | - | - | 0.52 | 0.08 | 1.28 |
| -Residual | - | - | - | - | 1.07 | 0.67 | 1.49 |
| **Females** |  |  |  |  |  |  |  |
| **Centrality** |  |  |  |  |  |  |  |
| -Density | 1.86 | 0.44 | 3.15 | 0.01 | - | - | - |
| -Density^2^ | -0.83 | -1.42 | -0.15 | 0.01 | - | - | - |
| -ID | - | - | - | - | 0.06 | 0.019 | 0.13 |
| -Residual | - | - | - | - | 0.03 | 0.02 | 0.04 |
| **Strength** |  |  |  |  |  |  |  |
| -Density | 1,743 | 1,406 | 2,076 | **<0.001** | - | - | - |
| -Density^2^ | -829.6 | -975.8 | -669.6 | **<0.001** | - | - | - |
| -ID | - | - | - | - | 2,066 | 661.6 | 4,465 |
| -Residual | - | - | - | - | 1,773 | 1,221 | 2,505 |
| **Degree** |  |  |  |  |  |  |  |
| -Density | 4.23 | -4.86 | 12.86 | <0.001 | - | - | - |
| -Density^2^ | -1.45 | -5.35 | 2.85 | 0.53 |  |  |  |
| -ID | - | - | - | - | 0.22 | 0.04 | 0.53 |
| -Residual | - | - | - | - | 1.23 | 0.84 | 1.65 |
