# Supplementary material for: Consistent individual differences and population plasticity in network-derived sociality: An experimental manipulation of density in a gregarious ungulate
Source: PLoS One. 2018 Mar 1;13(3):e0193425. doi: 10.1371/journal.pone.0193425 (PMC5832262; doi:10.1371/journal.pone.0193425)
Supplement: S5 Table — Values and ranks for graph strength (panels A and C for males and females, respectively) and eigenvector centrality (panels B and D for males and females, respectively) for each individual elk in our density treatments (low, medium, and high) and replicates (R1 and R2). Note, we did not include raw or ranked data for degree because there was little variation (see Results of main text). (DOCX) [file pone.0193425.s019.docx]

**Table S5**. Values and ranks for graph strength (panels A and C for males and females, respectively) and eigenvector centrality (panels B and D for males and females, respectively) for each individual elk in our density treatments (low, medium, and high) and replicates (R1 and R2). Note, we did not include raw or ranked data for degree because there was little variation (see Results of main text).

| **A)**  **ID** | **Graph Strength** | | | | | | **Rank** | | | | | |  |
| --- | --- | --- | --- | --- | --- | --- | --- | --- | --- | --- | --- | --- | --- |
|  | **Low**  **(R1)** | **Low**  **(R2)** | **Med**  **(R1)** | **Med**  **(R2)** | **High**  **(R1)** | **High (R2)** | **Low**  **(R1)** | **Low**  **(R2)** | **Med**  **(R1)** | **Med**  **(R2)** | **High**  **(R1)** | **High**  **(R2)** | **Mean Rank** |
| c14 | 36.39 | 28.17 | 64.65 | 21.72 | 293.36 | 160.26 | 10 | 10 | 8 | 11 | 3 | 6 | 8.0 |
| c19 | 93.01 | 42.00 | 85.47 | 105.78 | 84.71 | 75.74 | 4 | 8 | 5 | 2 | 11 | 10 | 6.7 |
| c24 | 38.90 | 41.28 | 72.05 | 44.31 | 181.22 | 112.95 | 9 | 9 | 7 | 9 | 8 | 8 | 8.3 |
| c40 | 96.91 | 46.71 | 88.62 | 104.44 | 89.07 | 75.90 | 3 | 7 | 4 | 3 | 10 | 9 | 6.0 |
| c49 | 56.99 | 83.86 | 60.27 | 60.94 | 221.00 | 144.88 | 6 | 6 | 10 | 8 | 6 | 7 | 7.2 |
| c8 | 62.15 | 128.30 | 128.21 | 70.04 | 274.36 | 209.54 | 5 | 2 | 3 | 6 | 4 | 3 | 3.8 |
| c80 | 56.11 | 108.83 | 73.24 | 104.39 | 197.86 | 181.01 | 7 | 3 | 6 | 4 | 7 | 4 | 5.1 |
| c81 | 49.83 | 100.42 | 61.69 | 64.00 | 222.50 | 171.64 | 8 | 4 | 9 | 7 | 5 | 5 | 6.3 |
| c82 | 7.020 | 20.86 | 37.23 | 33.79 | 97.36 | 58.993 | 11 | 11 | 11 | 10 | 9 | 11 | 10.5 |
| c83 | 102.52 | 100.17 | 160.56 | 76.49 | 443.36 | 217.60 | 1 | 5 | 2 | 5 | 2 | 2 | 2.8 |
| c84 | 97.41 | 231.59 | 195.67 | 149.21 | 480.36 | 311.40 | 2 | 1 | 1 | 1 | 1 | 1 | 1.2 |
| **B)** | **Eigenvector Centrality** | | | | | | **Rank** | | | | | |  |
| **ID** | **Low**  **(R1)** | **Low**  **(R2)** | **Med**  **(R1)** | **Med**  **(R2)** | **High**  **(R1)** | **High (R2)** | **Low**  **(R1)** | **Low**  **(R2)** | **Med**  **(R1)** | **Med**  **(R2)** | **High**  **(R1)** | **High**  **(R2)** | **Mean Rank** |
| c14 | 0.065 | 0.151 | 0.388 | 0.032 | 0.667 | 0.587 | 5 | 8 | 7 | 11 | 3 | 6 | 6.7 |
| c19 | 0.998 | 0.014 | 0.073 | 1.000 | 0.011 | 0.019 | 2 | 11 | 11 | 2 | 11 | 11 | 8.0 |
| c24 | 0.030 | 0.227 | 0.437 | 0.094 | 0.434 | 0.405 | 10 | 7 | 4 | 9 | 8 | 8 | 7.7 |
| c40 | 1.000 | 0.026 | 0.086 | 1.000 | 0.018 | 0.020 | 1 | 10 | 10 | 1 | 10 | 10 | 7.0 |
| c49 | 0.039 | 0.440 | 0.360 | 0.107 | 0.525 | 0.522 | 7 | 6 | 8 | 8 | 6 | 7 | 7.0 |
| c8 | 0.076 | 0.762 | 0.777 | 0.134 | 0.647 | 0.753 | 4 | 2 | 3 | 5 | 4 | 3 | 3.5 |
| c80 | 0.033 | 0.599 | 0.421 | 0.165 | 0.458 | 0.638 | 8 | 3 | 5 | 4 | 7 | 4 | 5.2 |
| c81 | 0.031 | 0.517 | 0.388 | 0.112 | 0.550 | 0.616 | 9 | 4 | 6 | 7 | 5 | 5 | 6.0 |
| c82 | 0.004 | 0.102 | 0.224 | 0.051 | 0.244 | 0.207 | 11 | 9 | 9 | 10 | 9 | 9 | 9.5 |
| c83 | 0.061 | 0.509 | 0.871 | 0.121 | 0.939 | 0.766 | 6 | 5 | 2 | 6 | 2 | 2 | 3.8 |
| c84 | 0.077 | 1.000 | 1.000 | 0.223 | 1.000 | 1.000 | 3 | 1 | 1 | 3 | 1 | 1 | 1.7 |
| **C)**  **ID** | **Graph Strength** | | | | | | **Rank** | | | | | |  |
|  | **Low**  **(R1)** | **Low**  **(R2)** | **Med**  **(R1)** | **Med**  **(R2)** | **High**  **(R1)** | **High (R2)** | **Low**  **(R1)** | **Low**  **(R2)** | **Med**  **(R1)** | **Med**  **(R2)** | **High**  **(R1)** | **High**  **(R2)** | **Mean Rank** |
| c26 | 169.0 | 64.67 | 215.16 | 216.97 | 111.13 | 58.12 | 2 | 5 | 5 | 5 | 3 | 8 | 4.7 |
| c27 | 96.25 | 44.50 | 218.79 | 18.91 | 65.97 | 40.53 | 10 | 7 | 4 | 12 | 9 | 9 | 8.5 |
| c28 | 122.30 | 76.33 | 168.75 | 212.96 | 97.82 | 66.95 | 7 | 4 | 9 | 7 | 4 | 6 | 6.2 |
| c29 | 109.12 | 113.17 | 213.61 | 236.60 | 77.51 | 73.46 | 9 | 2 | 6 | 4 | 6 | 3 | 5.0 |
| c30 | 134.04 | 29.00 | 154.14 | 154.23 | 60.25 | 39.20 | 6 | 10 | 10 | 9 | 10 | 10 | 9.2 |
| c31 | 168.21 | 127.00 | 244.82 | 297.87 | 132.71 | 85.36 | 3 | 1 | 2 | 1 | 1 | 1 | 1.5 |
| c33 | 177.09 | 95.50 | 235.41 | 296.67 | 84.92 | 63.93 | 1 | 3 | 3 | 2 | 5 | 7 | 3.5 |
| c34 | 121.42 | 39.83 | 200.32 | 214.56 | 72.83 | 69.26 | 8 | 8 | 7 | 6 | 7 | 4 | 6.7 |
| c35 | 39.47 | 8.33 | 72.23 | 154.02 | 11.11 | 16.78 | 12 | 11 | 11 | 10 | 12 | 12 | 11.3 |
| c37 | 151.18 | 61.33 | 254.48 | 268.15 | 119.22 | 67.58 | 4 | 6 | 1 | 3 | 2 | 5 | 3.5 |
| c39 | 53.93 | 6.83 | 37.83 | 34.53 | 44.620 | 22.17 | 11 | 12 | 12 | 11 | 11 | 11 | 11.3 |
| c69 | 143.42 | 38.50 | 186.47 | 203.81 | 71.11 | 84.71 | 5 | 9 | 8 | 8 | 8 | 2 | 6.7 |
| **D)** | **Eigenvector Centrality** | | | | | | **Rank** | | | | | |  |
| **ID** | **Low**  **(R1)** | **Low**  **(R2)** | **Med**  **(R1)** | **Med**  **(R2)** | **High**  **(R1)** | **High (R2)** | **Low**  **(R1)** | **Low**  **(R2)** | **Med**  **(R1)** | **Med**  **(R2)** | **High**  **(R1)** | **High**  **(R2)** | **Mean Rank** |
| c26 | 1.000 | 0.549 | 0.860 | 0.773 | 0.908 | 0.725 | 1 | 5 | 6 | 5 | 2 | 8 | 4.5 |
| c27 | 0.533 | 0.275 | 0.875 | 0.072 | 0.480 | 0.474 | 10 | 8 | 5 | 12 | 10 | 10 | 9.2 |
| c28 | 0.681 | 0.558 | 0.693 | 0.771 | 0.727 | 0.753 | 7 | 4 | 9 | 6 | 4 | 6 | 6.0 |
| c29 | 0.612 | 0.967 | 0.877 | 0.882 | 0.572 | 0.901 | 9 | 2 | 4 | 4 | 6 | 3 | 4.7 |
| c30 | 0.821 | 0.268 | 0.648 | 0.571 | 0.548 | 0.475 | 5 | 10 | 10 | 9 | 8 | 9 | 8.5 |
| c31 | 0.988 | 1.000 | 0.959 | 1.000 | 1.000 | 1.000 | 2 | 1 | 2 | 1 | 1 | 1 | 1.3 |
| c33 | 0.929 | 0.625 | 0.940 | 0.998 | 0.606 | 0.732 | 3 | 3 | 3 | 2 | 5 | 7 | 3.8 |
| c34 | 0.663 | 0.271 | 0.794 | 0.769 | 0.507 | 0.758 | 8 | 9 | 7 | 7 | 9 | 5 | 7.5 |
| c35 | 0.232 | 0.070 | 0.289 | 0.566 | 0.085 | 0.186 | 12 | 11 | 11 | 10 | 12 | 12 | 11.3 |
| c37 | 0.846 | 0.416 | 1.000 | 0.918 | 0.871 | 0.768 | 4 | 6 | 1 | 3 | 3 | 4 | 3.5 |
| c39 | 0.312 | 0.040 | 0.151 | 0.125 | 0.331 | 0.249 | 11 | 12 | 12 | 11 | 11 | 11 | 11.3 |
| c69 | 0.798 | 0.288 | 0.767 | 0.733 | 0.549 | 0.991 | 6 | 7 | 8 | 8 | 7 | 2 | 6.3 |
