# Supplementary material for: Consistent individual differences and population plasticity in network-derived sociality: An experimental manipulation of density in a gregarious ungulate
Source: PLoS One. 2018 Mar 1;13(3):e0193425. doi: 10.1371/journal.pone.0193425 (PMC5832262; doi:10.1371/journal.pone.0193425)
Supplement: S6 Table — Emboldened values indicate significant differences between metric values of the two replicates. (DOCX) [file pone.0193425.s020.docx]

**Table S6.** Results of Wilcoxon Rank Sum tests comparing network metric values of replicates 1 and 2 at each density for groups of male and female elk (Cervus canadensis) in Saskatchewan (2007). Emboldened values indicate significant differences between metric values of the two replicates.

| **Females** | | | |
| --- | --- | --- | --- |
| **Treatment** | **Metric** | **W** | **P-value** |
| low | Eigenvector centrality | 106 | **0.05** |
| medium | Eigenvector centrality | 81 | 0.63 |
| high | Eigenvector centrality | 62 | 0.59 |
| low | Graph strength | 124 | **0.002** |
| medium | Graph strength | 66 | 0.76 |
| high | Graph strength | 102 | 0.09 |
| low | Degree | 108 | **0.007** |
| medium | Degree | 102 | **0.02** |
| high | Degree | 60 | 0.17 |
| **Males** | | | |
| low | Eigenvector centrality | 36.5 | 0.12 |
| medium | Eigenvector centrality | 86.5 | 0.09 |
| high | Eigenvector centrality | 57.5 | 0.09 |
| low | Graph strength | 50 | 0.52 |
| medium | Graph strength | 72 | 0.48 |
| high | Graph strength | 87 | 0.09 |
| low | Degree | 53.5 | 0.66 |
| medium | Degree | 52 | 0.58 |
| high | Degree | 56.5 | 0.81 |
