# Supplementary material for: Consistent individual differences and population plasticity in network-derived sociality: An experimental manipulation of density in a gregarious ungulate
Source: PLoS One. 2018 Mar 1;13(3):e0193425. doi: 10.1371/journal.pone.0193425 (PMC5832262; doi:10.1371/journal.pone.0193425)
Supplement: S7 Table — Note, no significant relationship between network metrics calculated in different systems. (DOCX) [file pone.0193425.s021.docx]

**Table S7.** Summary of Pearson’s correlation coefficients comparing social network metrics derived from collars deployed on captive elk (this study) and wild elk [1]. Note, no significant relationship between network metrics calculated in different systems.

|  | **Graph strength** | **Eigenvector centrality** | **Degree** |
| --- | --- | --- | --- |
| High density | r = –0.14  t_24_ = –0.71  p = 0.48 | r = –0.31  t_24_ = –1.6  p = 0.11 | r = –0.19  t_24_ = –0.99  p = 0.32 |
| Medium density | r = 0.02  t_24_ = 0.10  p = 0.92 | r = 0.11  t_24_ = 0.56  p = 0.57 | r = –0.33  t_24_ = –1.7  p = 0.09 |
| Low density | r = –0.02  t_24_ = –0.09  p = 0.93 | r = –0.06  t_24_ = –0.31  p = 0.75 | r = –0.12  t_24_ = –0.59  p = 0.56 |

**References**

1. Vander Wal E, Paquet PC, Messier F, Mcloughlin PD. Effects of phenology and sex on social proximity in a gregarious ungulate. Can J Zool. 2013;91:601–9.
